# The impact of the new acute respiratory distress syndrome (ARDS) criteria on Berlin criteria ARDS patients: a multicenter cohort study

**DOI:** 10.1186/s12916-023-03144-7

**Published:** 2023-11-23

**Authors:** Lina Zhao, Fuhong Su, Nannan Zhang, Hening Wu, Yuehao Shen, Haiying Liu, Xuguang Li, Yun Li, Keliang Xie

**Affiliations:** 1 Department of Critical Care Medicine, Tianjin Medical University General Hospital, Tianjin, 300052 China; 2 Experimental Laboratory of Intensive Care, Université Libre de Bruxelles, 1000 Brussels, Belgium; 3 Department of Anesthesiology, Tianjin Institute of Anesthesiology, Tianjin Medical University General Hospital, Tianjin, 300052 China

**Keywords:** Acute respiratory distress syndrome, Multicenter cohort study, ARDS criteria

## Abstract

**Objective:**

The European Society of Intensive Care Medicine (ESICM) recently recommended changes to the criteria of acute respiratory distress syndrome (ARDS), patients with high-flow oxygen were included, however, the effect of these changes remains unclear. Our objectives were to evaluate the performance of these new criteria and to compare the outcomes of patients meeting the new ARDS criteria with those meeting the Berlin ARDS criteria.

**Methods:**

This was a retrospective cohort. The patients admitted to the intensive care unit (ICU) were diagnosed with ARDS. Patients were classified as meeting Berlin criteria ARDS (*n* = 4279), high-flow nasal oxygen (HFNO) criteria ARDS (*n* = 559), or new criteria ARDS (*n* = 4838).

**Results:**

In comparison with HFNO criteria ARDS and new criteria ARDS, patients with Berlin criteria ARDS demonstrated lower blood oxygen levels assessed by PaO_2_/FiO_2_, SpO_2_/FiO_2_, and ROX (SpO_2_/FiO_2_/respiratory rate) (*p* < 0.001); and higher severity of illness assessed by the Sequential Organ Failure Assessment (SOFA) score, Acute Physiology And Chronic Health Evaluations (APACHE II), Simplified Acute Physiology Score (SAPS II) (*p* < 0.001), (*p* < 0.001), and longer ICU and hospital stays (*p* < 0.001). In comparison with the HFNO criteria, patients meeting Berlin criteria ARDS had higher hospital mortality (10.6% vs. 16.9%; *p* = 0.0082), 28-day mortality (10.6% vs. 16.5%; *p* = 0.0079), and 90-day mortality (10.7% vs. 17.1%; *p* = 0.0083). ARDS patients with HFNO did not have severe ARDS; Berlin criteria ARDS patients with severe ARDS had the highest mortality rate (approximately 33%). PaO_2_/FiO_2_, SpO_2_/FiO_2_, and ROX negatively correlated with the SOFA and APACHE II scores. The SOFA and APACHE II scores had high specificity and sensitivity for prognosis in patients with new criteria ARDS.

**Conclusion:**

The new criteria of ARDS reduced the severity of illness, length of stay in the ICU, length of hospital stays, and overall mortality. SOFA and APACHE II scores remain important in assessing the prognosis of patients with new criteria ARDS.

**Trial registration:**

Registration number: ChiCTR2200067084.

**Supplementary Information:**

The online version contains supplementary material available at 10.1186/s12916-023-03144-7.

## Background

Acute respiratory distress syndrome (ARDS) is a clinical syndrome characterized by acute hypoxemic respiratory failure due to lung inflammation that is not caused by cardiogenic pulmonary edema. Over the last 55 years, ARDS criteria have focused primarily on the radiological appearance of the syndrome and the severity of the oxygenation defect (e.g., arterial oxygen tension (PaO_2_)/fraction of inspiration O_2_ (FiO_2_) ratio), which reflect both the original description of the syndrome [1] and its conceptual understanding [2]. The Berlin criteria [3], implies that the patient receives at least 5 cmH_2_O of positive end-expiratory pressure (PEEP) at the time of diagnosis. Patients who do not receive positive pressure cannot be considered to have ARDS.

The use of high-flow nasal oxygen (HFNO) has increased over the past decade, particularly during the COVID-19 pandemic [4]. Some COVID-19 patients not intubated and received HFNO also developed ARDS [5, 6], which is inconsistent with a previous diagnosis of ARDS. In addition, In addition, the Berlin criteria cause concern in the shortage of medical resources such as chest radiography, arterial blood gas measurements, and mechanical ventilation. The European Society of Intensive Care Medicine (ESICM) renewed criteria ARDS in July 2023 [7]. Patients receiving HFNO with a minimum flow rate of ≥ 30 L/min were included in the new criteria, even though they are not being ventilated with PEEP ≥ 5 cmH_2_O and use PaO_2_/FiO_2_ ≤ 300 mmHg or pulse oximeter oxygen saturation (SpO_2_)/FiO_2_ ≤ 315 (if SpO_2_ ≤ 97%) to identify hypoxemia and Ultrasound is added as an imaging modality to assess the condition of the lungs.

The impact of the new criteria of ARDS on patients with ARDS as criteria in Berlin is currently unclear; therefore, our aim was to determine the effect of the new ARDS criteria on patients meeting the Berlin criteria of ARDS using multicenter cohort studies and the performance of the new ARDS standard.

## Methods

### Data collection

This was a retrospective cohort. The data used in this study were extracted from the Department of Critical Care Medicine of the General Hospital of Tianjin Medical University, Medical Information Mart for Intensive Care IV (MIMIC-IV) database (v2.2) [8], and the eICU Collaborative Research Database (eICU-CRD) [9]. Data were collected from the Department of Critical Care Medicine of Tianjin Medical University General Hospital between December 2022 and April 2023. This study was approved by the Ethics Committee of Tianjin Medical University General Hospital (Project No: IRB2022-YX-222–01; registration number: ChiCTR2200067084). The eICU-CRD is a multicenter database of 335 units at 208 hospitals located throughout the USA between 2014 and 2015. Authors who acquired data from the databases completed the course and obtained certification (Record ID: 33690380). The MIMIC-IV consists of data from the Beth Israel Deaconess Medical Center from 2008 to 2019. The eICU-CRD and MIMIC-IV databases received ethical approval from the Institutional Review Boards and the Massachusetts Institute of Technology. As the three databases did not contain identified health information, a waiver of informed consent was included in the approval.

### Patients

All patients in the Tianjin Medical University General Hospital, MIMIC-IV, and eICU-CRD databases who met the following criteria were included in this study: (1) patients who were 18 years old or older; (2) patients diagnosed with ARDS, meeting the Berlin criteria [10] or the new criteria [7]; (3) patients without endotracheal intubation, HFNO ≥ 30 L/min, or under non-invasive ventilation (NIV)/continuous positive airway pressure (CPAP), PEEP ≥ 5 cmH_2_O; (4) hypoxemia: PaO_2_/FiO_2_ ≤ 300 mmHg or SpO_2_/FiO_2_ ≤ 315 mmHg with SpO_2_ ≤ 97%; and (6) chest x-ray and computed tomography (CT) show bilateral lung infiltrates. The exclusion criteria were as follows: (1) ICU stay or survival time of no more than 24 h; (2) congestive heart failure; (3) cardiogenic pulmonary edema; (4) A large areas of atelectasis; (5) alveolar hemorrhage; (6) massive pleural effusion; (7) pulmonary hypertension; (8) missing PaO_2_, FiO_2_, and SpO_2_ under NIV/CPAP; (9) missing PEEP values; and (10) patients without high flow or NIV/CPAP and missing data for more than 30% of variables were excluded from the variable selection process. According to the new diagnostic criteria, we divided patients with ARDS into a high-flow oxygen group and a Berlin criteria group. All patients admitted to the ICU of Tianjin Medical University General Hospital who were diagnosed with new ARDS were enrolled for screening. ARDS patients with MIMIC-IV and eICU-CRD databases were searched according to the new ARDS criteria.

### Data extraction

The clinical data of patients with MIMIC-IV and eICU-CRD ARDS were extracted using Structured Query Language (SQL) based on PostgreSQL tools (v10.0). The three databases of clinical variables included age, sex, coexisting illness (chronic pulmonary disease, immunosuppressive disease, liver disease, diabetes disease, renal disease, hypertension disease), coexisting illnesses were diagnosed based on the diagnostic ICD9 or ICD10 codes, and the worst of laboratory parameters within the first 24 h of confirmed ARDS (white blood cell count, hemoglobin, platelets, partial thromboplastin time, creatinine, and blood urea nitrogen), hemodynamic indicators within the first 24 h of confirmed ARDS (heart rate, systolic blood pressure, diastolic blood pressure, mean arterial pressure, and lactate levels). The patients’ prognostic scores included the Sequential Organ Failure Assessment (SOFA), Acute Physiology and Chronic Health Evaluation (APACHE II), and Simplified Acute Physiology Score II (SAPS II) within the first 24 h of confirmed ARDS. Patient prognosis included length of hospital stay, length of ICU stays, vasoactive drug use, hospital mortality, 28-day mortality, and 90-day mortality. Telephone follow-up was conducted for the enrolled patients, the last patient follow-up date was July 3, 2023, in Tianjin Medical University General Hospital. The follow-up information for patients with MIMIC-IV and eICU-CRD were searched in databases. The worst of breathing-related indicators included respiratory rate, PaO_2_, FiO_2_, SpO_2_, PaO_2_/FiO_2_, ratio of SpO_2_/FiO_2_ to respiratory rate (ROX), and SpO_2_/FiO_2_ during which ARDS was diagnosed.

### Statistical analysis

The Shapiro–Wilk test was used to detect continuous variables, which all had skewed distributions. Data are presented as proportions, medians, and interquartile ranges (25–75%). Comparisons among the high-flow oxygen group, Berlin criteria group, and the new ARDS criteria were made using the multiple chi-square and one-way analysis of variance. Differences between survivors and non-survivors were analyzed using the Wilcoxon rank-sum and Fisher tests. Kaplan–Meier estimates of hospital mortality, 28-day mortality, and 90-day mortality were performed for the high-flow oxygen and Berlin criteria groups. Differences were analyzed using the log-rank test. To identify independent predictors of the primary outcome, 28-day mortality, variables differing between survivors and non-survivors were entered into a multivariable regression model with a *p*-value < 0.05.

ARDS patients who received high-flow oxygen were compared with ARDS patients according to the Berlin criteria, severity of ARDS, and mortality using the ggalluvial and ggplot2 packages for visualization. ARDS patients received high-flow oxygen compared to ARDS patients with the Berlin criteria and ARDS patients with new criteria, as well as SOFA, APACHE II, or SAPS II. Scatterplot3d packages were used for visualization. In this study, the missing values were all less than 5%, and no treatment was performed on the missing values.

## Results

A total of 15,543 patients were included, the following patients were excluded: congestive heart failure (*n* = 4556), cardiogenic pulmonary edema (*n* = 1348), a large areas of atelectasis (*n* = 194), alveolar hemorrhage (*n* = 140), massive pleural effusion (*n* = 54), pulmonary hypertension (*n* = 89), missing blood oxygen-related indicators (*n* = 1858), age less than 18 years and survival or hospitalization < 24 h (*n* = 1768), there are more than 30% of patients with missing values (*n* = 698). After inclusion and exclusion criteria, a total of 559 patients with HFNO levels > 30 L/min and PaO_2_/FiO_2_ ≤ 300 or SpO_2_/FiO_2_ ratios ≤ 315 were diagnosed as HFNO ARDS. A total of 4279 patients met the ARDS Berlin diagnostic criteria, and a total of 4838 patients met the recently published criteria of ARDS (Additional file Fig. S1).

**Fig. 1:**
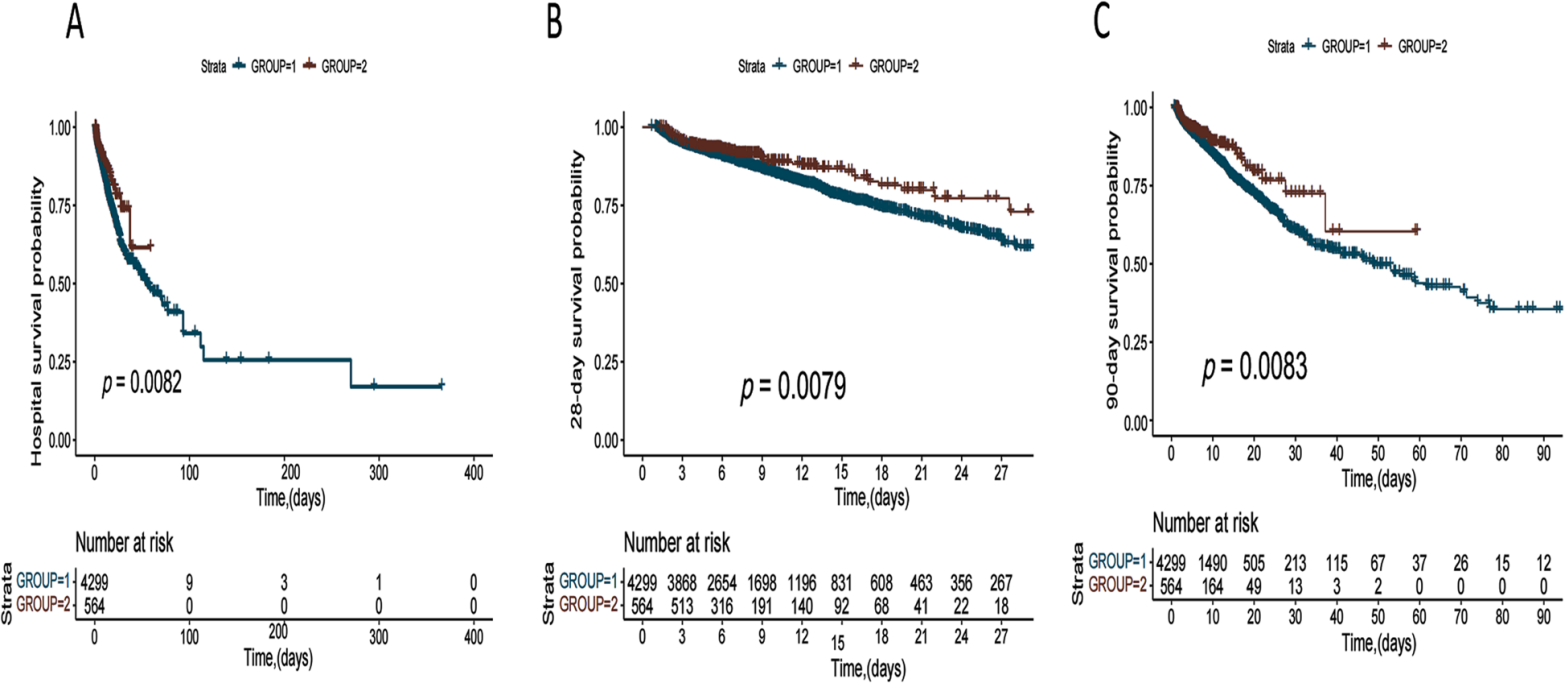
Kaplan–Meier estimates of survival by the acute respiratory distress syndrome (ARDS) subgroups. GROUP 1: Berlin criteria ARDS patients; GROUP 2: patients with high-flow nasal oxygenation (HFNO) criteria ARDS. Berlin criteria ARDS patient survival rates were lower than HFNO criteria ARDS patients, regarding overall mortality (**A**), 28-day mortality (**B**), and 90-day mortality (**C**)

Patients meeting the HFNO criteria were slightly younger (*p* < 0.001), more likely to had a history of immunosuppression disease (18.6%), and had a higher white blood cell count, and a lower platelet value and blood pressure level. Berlin defines patients with a lower SpO_2_/FiO_2_ [167.27 (118.00, 227.50)], PaO_2_/FiO_2_ [172.00 (120.00, 228.00)], and ROX index [6.90 (5.22, 8.59)]; more patients had moderate (51.6%) and severe (13.5%) ARDS. Berlin criteria patients with a worse prognosis as having higher SOFA scores (6.00 [4.00, 9.00]), longer hospital stays (7.32 [4.56, 12.83] and ICU stays (2.85 [1.66, 5.71]), and higher hospital mortality, 28-day mortality, and 90-day mortality. Despite our propensity matching scores, we found that patients with Berlin standard ARDS still had a worse prognosis. (Table 1, Fig. 1, and Additional file Table 1).

**Table 1:** Demographics for patients with acute respiratory distress syndrome (ARDS)

	High-flow oxygen ARDS (*n* = 559)	Berlin criteria ARDS (*n* = 4279)	New criteria ARDS (*n* = 4838)	*P*
Age	64.00 [51.00, 72.00]	65.00 [55.00, 75.00]	65.00 [55.00, 75.00]	< 0.001
Gender (male) (*n* (%))	205 (36.7)	2078 (48.6)	2283 (47.2)	< 0.001
**Coexisting illness, (*n* (%))**				
Chronic pulmonary disease	69 (12.3)	910 (21.3)	979 (20.2)	< 0.001
Immunosuppression disease	104 (18.6)	322 (7.5)	426 (8.8)	< 0.001
Liver disease	40 (7.2)	225 (5.3)	265 (5.5)	0.179
Diabetes disease	146 (26.1)	940 (22.0)	1086 (22.4)	0.087
Renal disease	64 (11.4)	817 (19.1)	881 (18.2)	< 0.001
Hypertension disease	102 (18.2)	1452 (33.9)	1554 (32.1)	< 0.001
**Laboratory parameters (mean (SD))/(median [IQR])**				
White blood cell count × 10ˆ^9^/L	15.50 [11.60, 19.90]	14.40 [10.80, 18.90]	14.60 [10.90, 19.00]	0.005
Hemoglobin(g/dL)	10.50 [9.00, 12.80]	10.20 [8.70, 12.10]	10.20 [8.80, 12.20]	0.002
Platelet (× 10ˆ9/L)	136.00 [99.50, 183.50]	161.00 [116.00, 216.00]	158.00 [113.00, 212.00]	< 0.001
International normalized ratio (median [IQR])	1.40 [1.20, 1.60]	1.40 [1.20, 1.60]	1.40 [1.20, 1.60]	0.341
Prothrombin time (median [IQR])	14.90 [13.20, 17.30]	15.30 [13.00, 17.50]	15.20 [13.00, 17.50]	0.644
Partial thromboplastin time (median [IQR])	31.60 [27.70, 40.60]	36.70 [29.30, 40.60]	35.65 [29.00, 40.60]	< 0.001
Creatinine (mg/dL)	1.00 [0.80, 1.38]	1.03 [0.80, 1.60]	1.00 [0.80, 1.60]	0.004
Blood urea nitrogen (mg/dL)	16.00 [12.00, 22.85]	20.00 [14.00, 31.00]	19.00 [14.00, 30.00]	< 0.001
**Hemodynamic indicators**				
Heart rate,bpm	98.00 [88.00, 114.00]	100.00 [90.00, 112.00]	100.00 [90.00, 112.00]	0.116
Systolic blood pressure, mmHg	92.00 [83.00, 105.00]	143.00 [95.00, 174.00]	136.00 [92.00, 170.00]	< 0.001
Diastolic blood pressure, mmHg	48.00 [42.00, 56.00]	72.00 [49.00, 89.00]	68.00 [47.00, 87.00]	< 0.001
Mean arterial pressure, mmHg	62.00 [55.00, 70.00]	97.00 [63.00, 117.00]	92.00 [61.00, 114.00]	< 0.001
Lactates (mmol/L)	2.30 [1.65, 3.10]	2.40 [1.70, 3.10]	2.40 [1.70, 3.10]	0.395
**Breathing-related indicators**				
Respiration rate, bpm	26.00 [23.00, 30.00]	21.00 [16.00, 25.00]	22.00 [16.00, 26.00]	< 0.001
ROX	8.00 [6.00, 11.00]	6.90 [5.22, 8.59]	7.93 [5.85, 10.80]	< 0.001
SpO_2_/FiO_2_	176.00 [140.00, 230.00]	167.27 [118.00, 227.50]	169.09 [122.12, 227.50]	< 0.001
PaO_2_/FiO_2_	180.00 [140.00, 234.00]	172.00 [120.00, 228.00]	173.00 [123.00, 229.00]	< 0.001
**ARDS severity (Berlin standard) (*n* (%))**				< 0.001
Mild	219 (39.2)	1493 (34.9)	1712 (35.4)	
Moderate	340 (60.8)	2210 (51.6)	2550 (52.7)	
Severe	0 (0.0)	576 (13.5)	576 (11.9)	
**Prognosis**				
Vasopressor (*n* (%))	66 (11.8)	801 (18.7)	867 (17.9)	< 0.001
SOFA	5.00 [3.00, 7.00]	6.00 [4.00, 9.00]	6.00 [4.00, 9.00]	< 0.001
Length of hospital stays, days (median [IQR])	6.61 [4.67, 11.00]	7.32 [4.56, 12.83]	7.21 [4.61, 12.54]	0.103
Length of ICU stays, days (median [IQR])	2.18 [1.33, 4.30]	2.85 [1.66, 5.71]	2.79 [1.58, 5.58]	< 0.001

*ROX* SpO_2_/FiO_2_/respiratory rate, *SOFA* Sequential Organ Failure Assessment, *PaO*_*2*_ arterial oxygen tension, *FiO*_*2*_ fraction of inspiration O_2_, *SpO*_*2*_ pulse oximeter oxygen saturation, *ARDS* acute respiratory distress syndrome

**Fig. 2:**
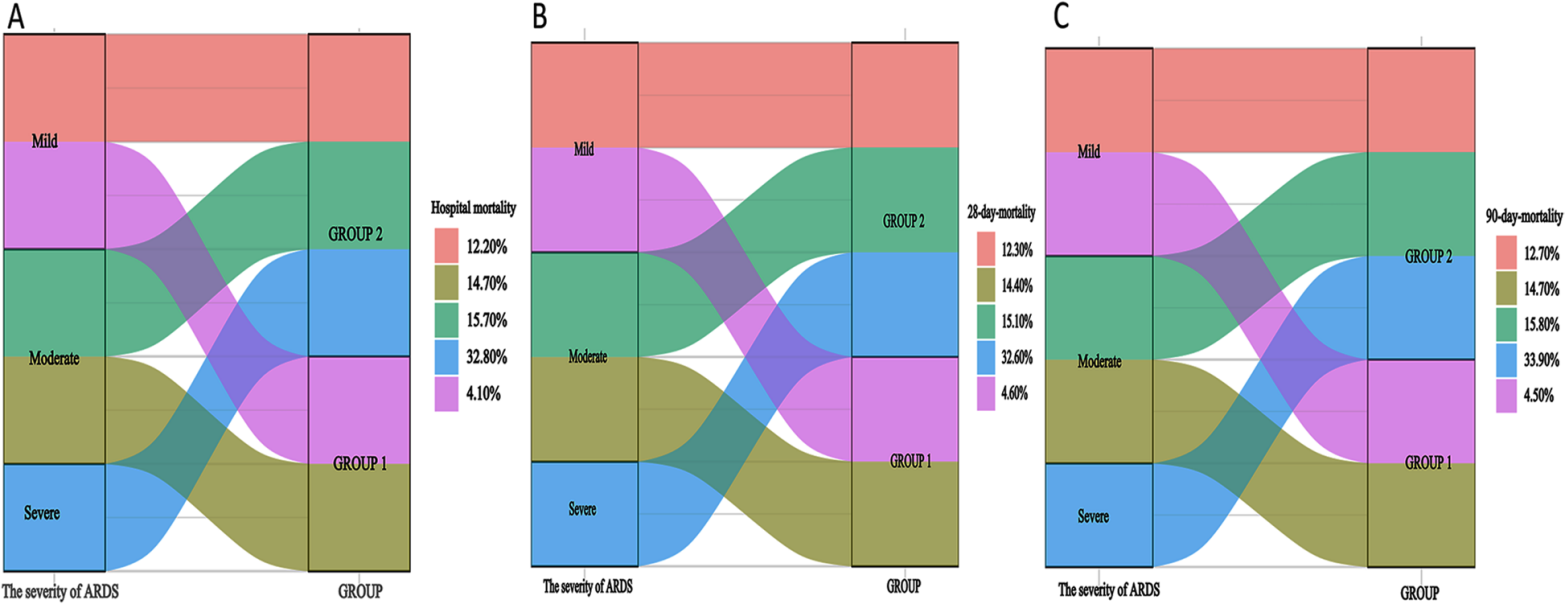
Sankey diagram plot of the relationship between acute respiratory distress syndrome (ARDS) subtypes and ARDS severity and mortality. GROUP 1: Berlin criteria ARDS patients; GROUP 2: high-flow nasal oxygenation (HFNO) criteria ARDS patients. HFNO criteria ARDS patients do not have severe ARDS. Berlin criteria ARDS patients have severe ARDS with hospitalization mortality (**A**), 28-day mortality (**B**), and 90-day mortality (**C**) rates of 32.80%, 32.60%, and 33.90%, respectively

Stratified analysis of disease severity in patients with ARDS, in ARDS patients with HFNO, none had no severe ARDS, and 39.2% and 60.8% accounted for mild and moderate ARDS. ARDS patients with Berlin diagnostic criteria had more severe ARDS. ARDS patients with severe ARDS in the Berlin diagnostic criteria had the highest in-hospital mortality (32.8%), 28-day mortality (32.6%), and 90-day mortality (33.9%) (Fig. 2).

The data from the three databases (General Hospital of Tianjin Medical University, MIMIC IV, and eICU) were divided into survival and non-survival groups according to the 28-day and 90-day mortality. The data from the three databases showed that PaO_2_/FiO_2_, SpO_2_/FiO_2_, ROX, and SpO_2_ in the non-survival group were significantly lower than those in the survival group (*p* < 0.001), whereas the non-survival groups had more severe ARDS and higher FiO_2_, APACHE II, SAPS II, and SOFA scores (*p* < 0.001) (Table 2, Additional files Table 2–7). SpO_2_/FiO_2_, lactate, and SOFA score were independent risk factors for the 28-day mortality in patients with new criteria ARDS (Additional file Table 8).

**Table 2:** Comparison of oxygenation indexes in surviving patients and non-surviving patients with acute respiratory distress syndrome (ARDS) in three cohorts

	28-day survival group	Non-28-day survival group	*P*-value	90-day survival group	Non-90-day survival group	*P*-value
Data from the General Hospital of Tianjin Medical University	*n* = 258	*n* = 87		*n* = 250	*n* = 95	
SpO_2_/FiO_2_ (median [IQR])	173.16 [145.13, 194.00]	144.26 [93.00, 172.73]	< 0.001	174.55 [145.13, 194.00]	146.67 [93.50, 172.73]	< 0.001
ROX	6.70 [4.82, 8.50]	5.00 [3.45, 7.25]	< 0.001	6.70 [4.90, 8.50]	4.90 [3.45, 7.35]	< 0.001
Berlin criteria ARDS	164 (63.6)	79 (90.8)	< 0.001	156 (62.4)	87 (91.6)	< 0.001
The severity of ARDS			< 0.001			< 0.001
Mild	125 (48.4)	18 (20.7)		123 (49.2)	20 (21.1)	
Moderate	98 (38.0)	41 (47.1)		95 (38.0)	44 (46.3)	
Severe	35 (13.6)	28 (32.2)		32 (12.8)	31 (32.6)	
APACHE II (median [IQR])	16.00 [13.00, 20.00]	23.00 [18.00, 28.00]	< 0.001	18.00 [13.00, 22.00]	21.00 [16.00, 25.00]	0.001
SOFA (median [IQR])	6.00 [4.00, 9.00]	12.00 [8.00, 16.00]	< 0.001	6.00 [4.00, 8.75]	12.00 [8.00, 16.00]	< 0.001
Data from the EICU database	*n* = 2168	*n* = 516		*n* = 2149	*n* = 535	
SpO_2_/FiO_2_ (median [IQR])	182.00 [140.00, 230.00]	153.33 [100.00, 190.00]	< 0.001	182.00 [140.00, 230.00]	153.33 [100.00, 190.00]	< 0.001
ROX	9.65 [7.10, 12.30]	8.00 [6.30, 11.00]	< 0.001	9.70 [7.10, 12.30]	8.00 [6.30, 11.00]	< 0.001
Berlin criteria ARDS	2131 (98.3)	507 (98.3)	1000	2112 (98.3)	526 (98.3)	1.000
The severity of ARDS (*n* (%))			< 0.001			< 0.001
Mild	865 (39.9)	146 (28.3)		862 (40.1)	149 (27.9)	
Moderate	1075 (49.6)	247 (47.9)		1063 (49.5)	259 (48.4)	
Severe	228 (10.5)	123 (23.8)		224 (10.4)	127 (23.7)	
SAPS II (median [IQR])	54.00 [33.00, 72.00]	48.00 [30.00, 73.00]	0.009	54.00 [33.00, 71.00]	49.00 [30.00, 73.00]	0.017
SOFA (median [IQR])	7.00 [5.00, 9.00]	11.00 [8.00, 14.00]	< 0.001	7.00 [5.00, 9.00]	11.00 [8.00, 14.00]	< 0.001
Data from the MIMIC IV database	*n* = 1648	*n* = 161		*n* = 1646	*n* = 163	
SpO_2_/FiO_2_ (median [IQR])	168.00 [117.00, 222.50]	136.67 [97.00, 186.00]	< 0.001	168.00 [117.00, 222.50]	136.00 [97.67, 183.00]	< 0.001
ROX	6.45 [4.62, 8.54]	4.92 [3.18, 7.25]	< 0.001	6.46 [4.63, 8.54]	4.88 [3.18, 7.10]	< 0.001
Berlin criteria ARDS	1279 (77.6)	119 (73.9)	0.332	1278 (77.6)	120 (73.6)	0.284
The severity of ARDS (*n* (%))			< 0.001			< 0.001
Mild	528 (32.0)	30 (18.6)		528 (32.1)	30 (18.4)	
Moderate	990 (60.1)	87 (54.0)		989 (60.1)	88 (54.0)	
Severe	130 (7.9)	44 (27.3)		129 (7.8)	5 (27.6)	
SAPS II (median [IQR])	31.00 [25.00, 39.00]	47.00 [37.00, 60.00]	< 0.001	31.00 [25.00, 39.00]	47.00 [37.00, 59.50]	< 0.001
SOFA (median [IQR])	4.00 [3.00, 6.00]	8.00 [5.00, 12.00]	< 0.001	4.00 [3.00, 6.00]	8.00 [5.00, 12.00]	< 0.001

*ROX* SpO_2_ /FiO_2_/respiratory rate, *SOFA* Sequential Organ Failure Assessment, *PaO*_*2*_ arterial oxygen tension, *FiO*_*2*_ fraction of inspiration O_2_, *SpO*_*2*_ pulse oximeter oxygen saturation, *SAPS II* Simplified Acute Physiology Score II, *APACHE II* acute physiology and chronic health evaluation, *ARDS* acute respiratory distress syndrome

**Fig. 3:**
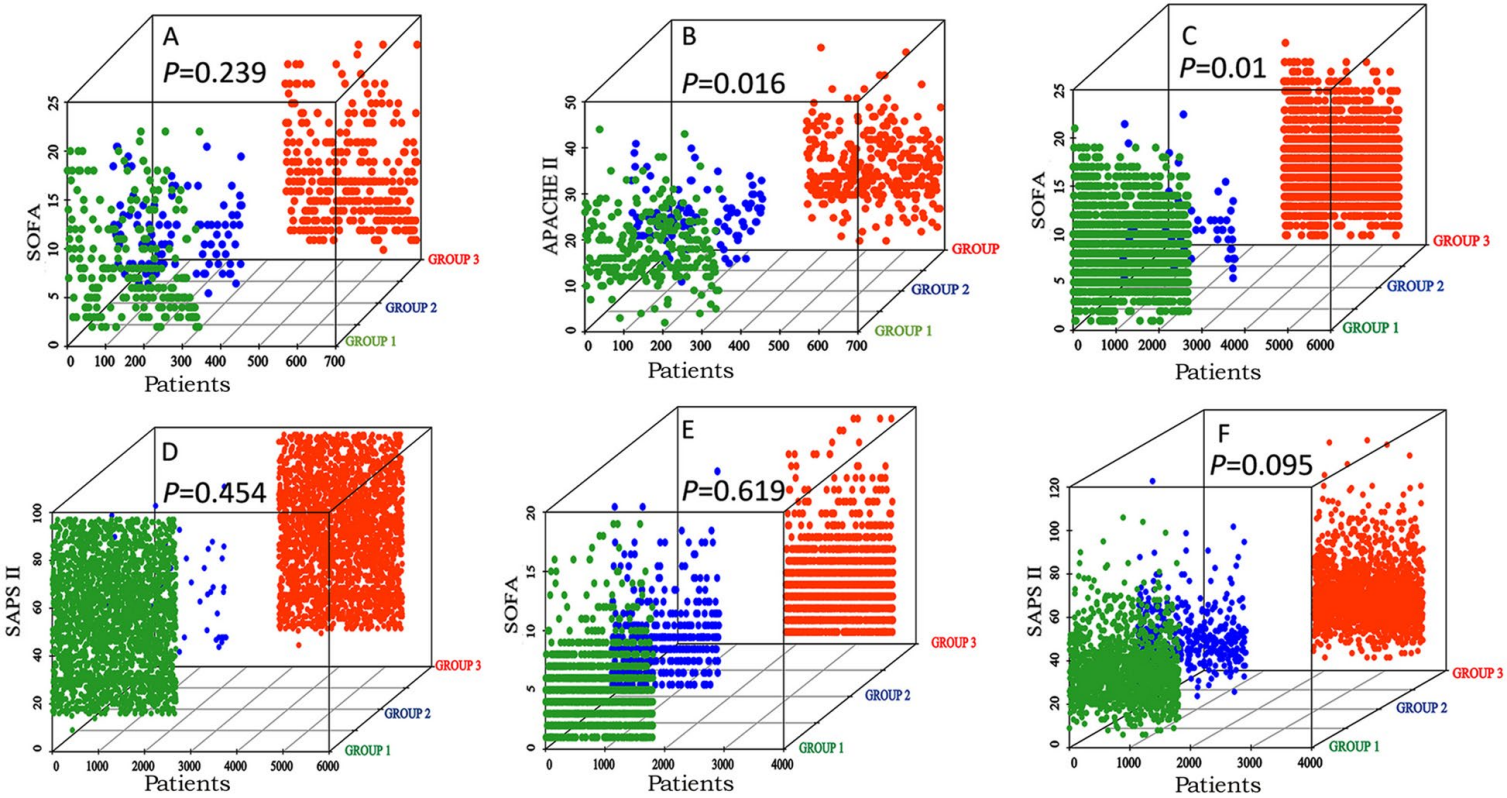
The relationship between acute respiratory distress syndrome (ARDS) subtype and Sequential Organ Failure Assessment (SOFA) score, Acute Physiology and Chronic Health Evaluation (APACHE II), and Simplified Acute Physiology Score (SAPS II). GROUP 1: Berlin defines ARDS patients; GROUP 2: Patients with high flow oxygenated ARDS; GROUP 3: Newly criteria ARDS. **A** and **B** show the relationship between the ARDS subtype and the SOFA score and APACHE II score in the data from Tianjin Medical University General Hospital. The Berlin criteria SOFA score and APACHE II scores were higher than those of patients with HFNO and new criteria ARDS. **C** and **D** show the relationship between the ARDS subtype and the SOFA score and SAPS II score in the data of the eICU database. The SOFA score and SAPS II of Berlin criteria ARDS patients were higher than in patients with HFNO criteria ARDS in the eICU database. **E** and **F** show the relationship between the ARDS subtype and the SOFA score and SAPS II score in the data from the Medical Information Mart for Intensive Care IV (MIMIC-IV) database. The SAPS II score of Berlin criteria ARDS patients was higher than patients with NHNO criteria ARDS in the MIMIC-IV database

Patients with ARDS in the three databases were divided into three groups: Berlin criteria ARDS (GROUP 1), HFNO ARDS (GROUP 2), and new criteria ARDS (GROUP 3). Regarding ARDS patients in the General Hospital of Tianjin Medical University, the SOFA and APACHE II of GROUP 1 were higher than GROUP 2 and GROUP 3 (Fig. 3A, B). In patients with ARDS in the eICU database and MIMIC-IV database, the SOFA and SAPS II of GROUP 1 were higher than GROUP 2 (Fig. 3C–F) (Additional file Table 9). The SOFA score identified that the area under the curve (AUC) for the mortality rate in the new criteria ARDS patients was 0.769–0.808, the specificity was 73.3–84.0%, and the sensitivity was 55.8–67.1%. The APACHE II score identified that the AUC for the mortality rate of the newly criteria ARDS patients was 0.758–0.776, the specificity was 73.9–78.3%, and the sensitivity was 62.1–65.8%. The SAPS II score identified that the AUC for the mortality rate of the newly criteria ARDS patients was 0.578–0.580, the specificity was 56.8%, and the sensitivity was 65.8–65.9% (Additional file Fig. S2).

**Fig. 4:**
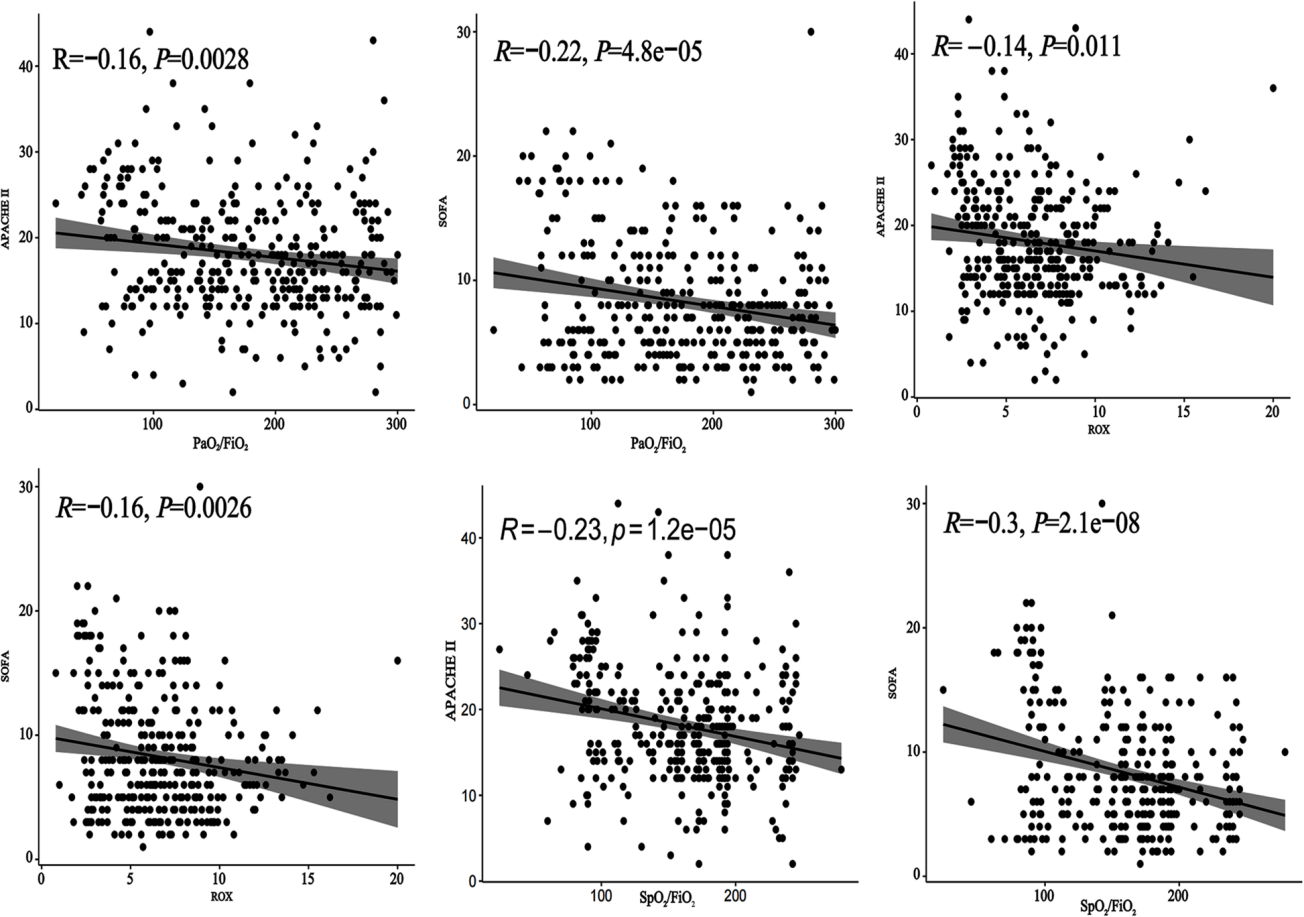
The relationship between oxygenation indicators and Sequential Organ Failure Assessment (SOFA) scores and the Acute Physiology and Chronic Health Evaluation (APACHE II). PaO_2_/FiO_2_, SpO_2_/FiO_2_, and ROX were negatively correlated with SOFA and APACHE II

The correlations between the blood oxygen indices PaO_2_/FiO_2_, ROX, and SpO_2_/FiO_2_, and the SOFA, APACHE II, and SAPS II in the three databases were analyzed. Figure 4 shows that PaO_2_/FiO_2_, ROX, and SpO_2_/FiO_2_ were negatively correlated with the SOFA and APACHE II scores in the Department of Critical Care Medicine of Tianjin Medical University General Hospital for new criteria ARDS patients. PaO_2_/FiO_2_, SpO_2_/FiO_2_, and ROX were negatively correlated with SOFA and SAPS II in eICU database and MIMIC IV database (Additional file Fig. S3–Fig. S4).

## Discussion

The main findings of the current study include (1) the new criteria had led to an increase in the incidence of ARDS; (2) patients with the new ARDS diagnostic criteria had a better prognosis than those with ARDS in the Berlin diagnostic criteria; and (3) ROX index, SpO_2_/FiO_2_, and PaO_2_/FiO_2_, SOFA, and APACHE II scores played important in the assessing the severity of ARDS and the prognosis of new criteria ARDS.

The results of this study show that the number of new criteria for ARDS patients was more than those for the Berlin diagnostic criteria. With the global epidemic of the novel coronavirus, the expansion of diagnostic criteria in Berlin criteria has been further promoted, these include non-endotracheal intubated with ARDS patients, endotracheal intubated with ARDS patients, and resource-limited with ARDS patients. The new criteria of ARDS facilitate the identification of patients with ARDS in the early stages to help clinicians intervene early in patients, the intervention of early clinicians becomes more feasible, which is beneficial to reduce the mortality rate of patients with ARDS. The new diagnostic criteria for ARDS may still need to be validated in clinical practice, including assessments of reliability, feasibility, and prognostic effectiveness. In addition, our cohort study demonstrated that the mortality rate in Berlin criteria patients with severe Berlin ARDS criteria was about 33%, the results of the study showed that the prognosis of patients with ARDS was poor. The past decade has demonstrated that patients with ARDS still have a high mortality rate (approximately 40%) [11, 12], including the Berlin ARDS criteria standard of 2012 [3, 10]. The outcomes of this study were consistent with those of previous studies [11, 12]. The results of this study show that the highest mortality rate in patients with HFNO ARDS was less than 15%. The ESICM had expanded the Berlin criteria of ARDS to include HFNO patients to ensure the early and timely detection of ARDS patients [7]. The new criteria of ARDS and, consequently, the severity of the illness and risk of mortality have been potentially underappreciated following the translation of these guidelines into clinical practice.

This study compared the Berlin criteria oxygenation index and showed that the new criteria and HFNO criteria ARDS patients had a higher ROX index, SpO_2_/FiO_2_, and PaO_2_/FiO_2_. Compared to the Berlin criteria, the new criteria and HFNO criteria ARDS patients had milder lung conditions. Multiple studies have shown that the ROX index, SpO_2_/FiO_2_, and PaO_2_/FiO_2_ were important indicators to assess the severity of ARDS [13–16]. One of the most interesting aspects of this study is S_P_O_2_/FiO_2_, this update to the new definition agrees to the use of S_P_O_2_/FiO_2_ as an alternative to PaO_2_/FiO_2_ for diagnosing ARDS, recent ARDS clinical trials had used S_P_O_2_/FiO_2_ for patient selection, and patients diagnosed with ARDS by S_P_O_2_/FiO_2_ had similar clinical outcomes to patients diagnosed with blood gas analysis [15, 16], the committee agreed to use the Rice linear equation to define the cut-off values for S_P_O_2_/FiO_2_, because its sensitivity and specificity for hypoxemia were almost consistent with nonlinear estimates and were simpler to calculate [7]. Our findings reaffirm that S_P_O_2_/FiO_2_ plays the same important role as PaO_2_/FiO_2_ in diagnosing and assessing the severity of ARDS. Therefore, among the new ARDS diagnostic criteria, the ROX index, SpO_2_/FiO_2_, and PaO_2_/FiO_2_ ratios are still the indicators that we need to focus on.

The SOFA, APACHE II, and SAPS II scores were important for assessing the prognosis of critically ill patients [17–20]. The results of this cohort study showed that the SOFA, APACHE II, and SAPS II scores of patients with HFNO criteria ARDS were lower than those of patients with Berlin criteria ARDS. Furthermore, patients with HFNO criteria ARDS had low mortality. This is consistent with the results of other prognostic indicators in this study. Further correlation analysis showed that the SOFA and APACHE II scores were negatively correlated with ROX, SpO_2_/FiO_2_, and PaO_2_/FiO_2_ in the new criteria ARDS, which is consistent with previous Berlin criteria studies [21, 22]. Schmidt et al. found that the SOFA score was the independent risk of death in patients with Berlin ARDS at 90 days [23]. Sinha P et al. found that APACHE II was associated with the hyperinflammatory response of Berlin criteria ARDS, which affected the prognosis of patients with ARDS [24]. However, this study found that the SOFA and APACHE II scores had high AUC values, specificity, and sensitivity for identifying the mortality rate of patients with new criteria ARDS. This shows that the SOFA score and APACHE II still play important roles in the prognostic assessment of newly diagnosed patients with ARDS. The relationship between SOFA, APACHE II and the new ARDS diagnostic criteria subtypes needs to be further investigated in the future.

Our study had several limitations. First, our criteria of ARDS are newly updated according to the ESICM guidelines, as follows: radiographic changes in the lungs identified by chest x-ray and chest CT, patients with HFNO ≥ 30 L/min or under NIV/CPAP, PEEP ≥ 5 cmH_2_O, and hypoxia criteria according to PaO_2_/FiO_2_ ≤ 300 mmHg or SpO_2_/FiO_2_ ≤ 315 mmHg with SpO_2_ ≤ 97%. We did not use an ultrasound evaluation of the lungs to confirm pulmonary manifestations in patients with underlying ARDS, which may have resulted in some patients with ARDS not being properly identified. Second, although this study excluded congestive heart failure, cardiogenic pulmonary edema, large-scale infarction, and other diseases, there may be other mixed diseases that affect hypoxia, which may have contributed to a selection bias in the cohort of patients with ARDS. Thirdly, despite the above limitations, this study has some clinical value in preliminarily exploring the impact of the updated criteria of ARDS on the prognostic assessment of patients with Berlin criteria ARDS through a multicenter cohort study. Finally, the data of the study from three different databases and was different time periods, which may led to heterogeneity, although we applied the same diagnostic criteria.

## Conclusion

This cohort study found that patients with new ARDS criteria had a better prognosis than those with Berlin criteria ARDS. ROX, SpO_2_/FiO_2_, and PaO_2_/FiO_2_ can be used to assess the severity of ARDS disease and still play important roles in new ARDS criteria. The SOFA and APACHE II scores play an important role in assessing the prognosis of patients with new ARDS criteria.

## Supplementary Information

**Additional file 1: Additional file Fig. S1.** Flow chart for patient selection. **Additional file table 1.** Demographics for patients with ARDS with Propensity match score. **Additional file table 2.** The 28-day prognosis analysis of patients with new ARDS was analyzed from the General Hospital of Tianjin Medical University. **Additional file table 3.** The 90-day prognosis analysis of patients with new ARDS was analyzed from the General Hospital of Tianjin Medical University. **Additional file table 4.** The 28-day prognosis analysis of patients with new ARDS was analyzed from the eICU database. **Additional file table 5.** The 90-day prognosis analysis of patients with new ARDS was analyzed from the eICU database. **Additional file table 6.** The 28-day prognosis analysis of patients with new ARDS was analyzed from the MIMIC IV database. **Additional file table 7.** The 90-day prognosis analysis of patients with new ARDS was analyzed from the MIMIC IV database. **Additional file table 8.** Univariate and multivariate analysis of 28-day survival in patients with ARDS in three database. **Additional file table 9.** Comparison of SOFA scores, APACHE II scores, and SAPS II scores in databases and new and Berlin ARDS diagnostic criteria. **Additional file Fig. S2.** The SOFA score and the APACHE II、SAPS II score predict the ROC curve the prognosis of newly criteria ARDS patients. **Additional file Fig. S3.** The relationship between oxygenation indicators and SOFA and SAPS II in eICU database. **Additional file Fig. S4.** The relationship between oxygenation indicators and SOFA and SAPS II in MIMIC IV database. **Additional file Fig. S5.** Multivariate correlation analysis plot. **Additional file Fig. S6.** Missing values for variables in the data. **Additional file Fig. S7.** The distribution of variables in the data. **Additional file table 10.** Direction of abnormal values and distribution transformation.

## Data Availability

If the reason is reasonable, the original data can be requested from the corresponding author.

## Abbreviations

APACHE II: Acute Physiology and Chronic Health Evaluation
ARDS: Acute respiratory distress syndrome
CPAP: Continuous positive airway pressure
eICU-CRD: EICU Collaborative Research Database
ESICM: European Society of Intensive Care Medicine
FiO_2_: Fraction of inspiration O_2_
HFNO: High-flow nasal oxygen
ICU: Intensive care unit
MIMIC-IV: Medical Information Mart for Intensive Care IV
NIV: Non-invasive ventilation
PaO_2_: Arterial oxygen tension
PEEP: Positive end-expiratory pressure
ROX: Ratio of SpO_2_/FiO_2_ to respiratory rate
SAPS II: Simplified Acute Physiology Score
SOFA: Sequential Organ Failure Assessment
SpO_2_: Pulse oximeter oxygen saturation
SQL: Structured Query Language
