# Correction: The impact of the new acute respiratory distress syndrome (ARDS) criteria on Berlin criteria ARDS patients: a multicenter cohort study

**DOI:** 10.1186/s12916-024-03750-z

**Published:** 2024-11-09

**Authors:** Lina Zhao, Fuhong Su, Nannan Zhang, Hening Wu, Yuehao Shen, Haiying Liu, Xuguang Li, Yun Li, Keliang Xie

**Affiliations:** 1 Department of Critical Care Medicine, Tianjin Medical University General Hospital, Tianjin, 300052 China; 2 Experimental Laboratory of Intensive Care, Université Libre de Bruxelles, 1000 Brussels, Belgium; 3 Department of Anesthesiology, Tianjin Institute of Anesthesiology, Tianjin Medical University General Hospital, Tianjin, 300052 China

**Correction** **:** **BMC Med 21, 456 (2023)**

**https://doi.org/10.1186/s12916-023-03144-7**

The original article [1] displays two errors which the authors wish to clarify:

Firstly, there is an inconsistency between the number of patients in GROUP 1 and GROUP 2 in Fig. 1; instead, GROUP1 should state 4279 patients, and GROUP2 should state 559 patients.

Secondly, GROUP1 and GROUP2 had inverted descriptions in the caption of Fig 2; therefore, the caption should instead read as follows:

'GROUP 1: high-fow nasal oxygenation (HFNO) criteria ARDS patients; GROUP 2: Berlin criteria ARDS patients.'

The authors apologise for these oversights and are thankful for understanding and assistance in this matter.
