# Supplementary material for: The impact of the new acute respiratory distress syndrome (ARDS) criteria on Berlin criteria ARDS patients: a multicenter cohort study
Source: BMC Med. 2023 Nov 23;21:456. doi: 10.1186/s12916-023-03144-7 (PMC10666384; doi:10.1186/s12916-023-03144-7)
Supplement: Supplementary file 1 — Additional file 1: Additional file Fig. S1. Flow chart for patient selection. Additional file table 1. Demographics for patients with ARDS with Propensity match score. Additional file table 2. The 28-day prognosis analysis of patients with new ARDS was analyzed from the General Hospital of Tianjin Medical University. Additional file table 3. The 90-day prognosis analysis of patients with new ARDS was analyzed from the General Hospital of Tianjin Medical University. Additional file table 4. The 28-day prognosis analysis of patients with new ARDS was analyzed from the eICU database. Additional file table 5. The 90-day prognosis analysis of patients with new ARDS was analyzed from the eICU database. Additional file table 6. The 28-day prognosis analysis of patients with new ARDS was analyzed from the MIMIC IV database. Additional file table 7. The 90-day prognosis analysis of patients with new ARDS was analyzed from the MIMIC IV database. Additional file table 8. Univariate and multivariate analysis of 28-day survival in patients with ARDS in three database. Additional file table 9. Comparison of SOFA scores, APACHE II scores, and SAPS II scores in databases and new and Berlin ARDS diagnostic criteria. Additional file Fig. S2. The SOFA score and the APACHE II、SAPS II score predict the ROC curve the prognosis of newly criteria ARDS patients. Additional file Fig. S3. The relationship between oxygenation indicators and SOFA and SAPS II in eICU database. Additional file Fig. S4. The relationship between oxygenation indicators and SOFA and SAPS II in MIMIC IV database. Additional file Fig. S5. Multivariate correlation analysis plot. Additional file Fig. S6. Missing values for variables in the data. Additional file Fig. S7. The distribution of variables in the data. Additional file table 10. Direction of abnormal values and distribution transformation. [file 12916_2023_3144_MOESM1_ESM.docx]

**Additional file Fig. S1:Flow chart for patient selection**


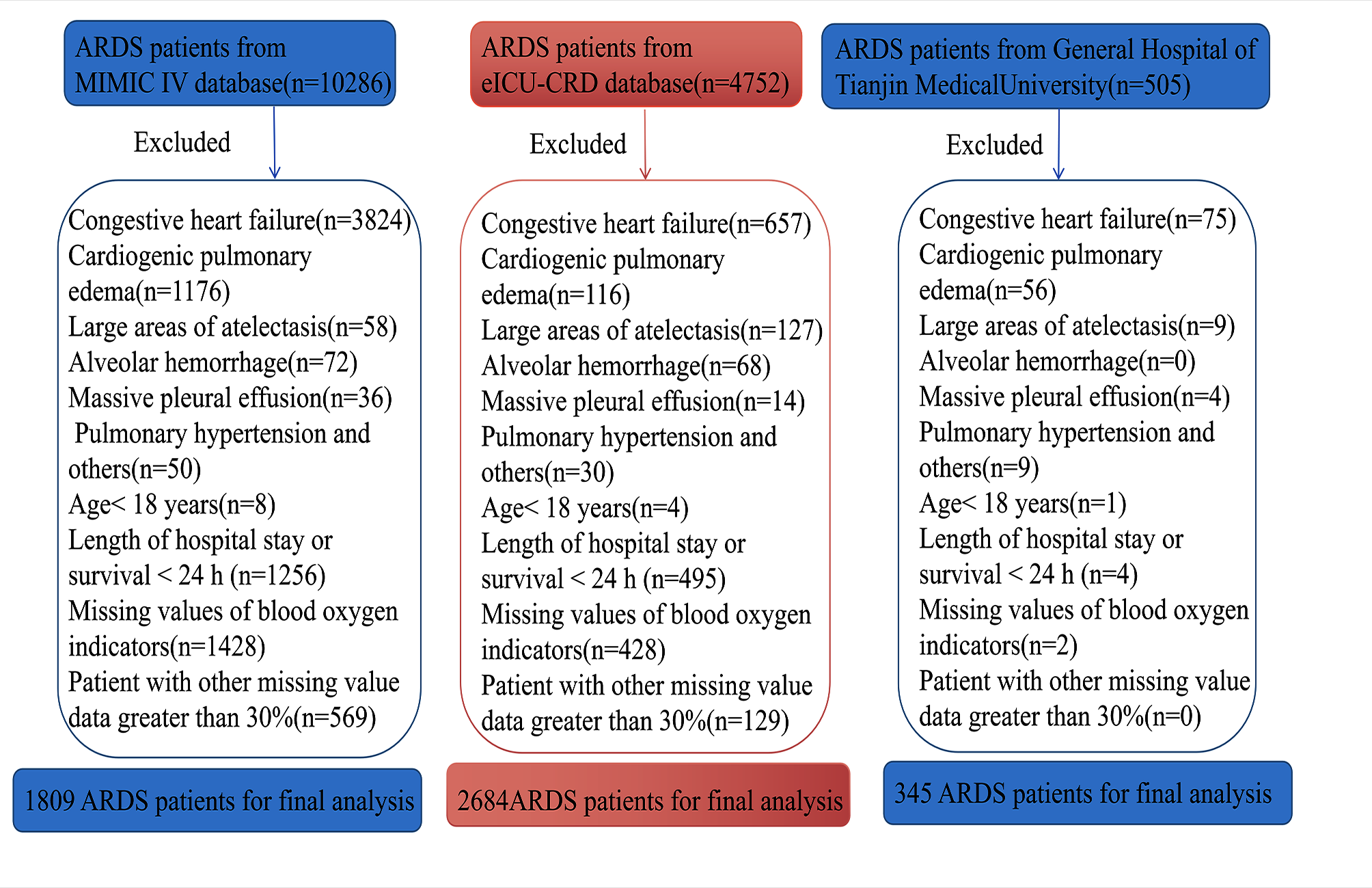


ARDS: Acute respiratory distress syndrome; MIMIC IV: Medical Information Mart for Intensive Care IV;eICU-CRD:eICU Collaborative Research Database.

| **Additional file table 1** Demographics for patients with ARDS with Propensity match score | | | |
| --- | --- | --- | --- |
|  | High-flow oxygen ARDS（*n*=555） | Berlin criteria ARDS  (*n*=555) | *P* |
| Age | 64.00 [51.00, 72.00] | 65.00 [53.00, 73.00] | 0.339 |
| Gender(male),(n (%)) | 204 (36.8) | 189 ( 34.1) | 0.38 |
| **Coexisting illness, (n (%))** |  |  |  |
| Chronic.pulmonary.disease | 69 (12.4) | 60 ( 10.8) | 0.454 |
| Immunosuppression. disease | 103 (18.6) | 45 ( 8.1) | <0.001 |
| Liver.disease | 40 ( 7.2) | 33 ( 5.9) | 0.468 |
| Diabetes.disease | 144 (25.9) | 168 ( 30.3) | 0.125 |
| Renal.disease | 64 (11.5) | 108 ( 19.5) | <0.001 |
| Hypertension.disease | 102 (18.4) | 276 ( 49.7) | <0.001 |
| **Laboratory parameters(mean (SD))/(median [IQR])** | | | |
| White blood cell count x 10ˆ^9^/L | 15.40 [11.60, 19.90] | 15.00 [10.80, 19.50] | 0.096 |
| Hemoglobin(g/dL) | 10.50 [9.00, 12.80] | 13.30 [9.70, 70.00] | <0.001 |
| Platelet (×10ˆ9 /L) | 136.00 [99.50, 183.50] | 129.00 [79.00, 173.00] | 0.003 |
| International Normalized Ratio(median [IQR]) | 1.40 [1.20, 1.60] | 1.40 [1.22, 1.50] | 0.925 |
| ProthrombinTime(median [IQR]) | 14.90 [13.20, 17.30] | 14.80 [13.40, 16.80] | 0.68 |
| Partial Thromboplastin Time (median [IQR]) | 31.60 [27.70, 40.50] | 33.40 [28.40, 42.00] | 0.016 |
| Creatinine(mg/dL) | 1.00 [0.80, 1.38] | 1.11 [0.80, 2.20] | <0.001 |
| Blood urea nitrogen (mg/dL) | 16.00 [12.00, 22.85] | 16.80 [10.60, 24.00] | 0.488 |
| **Hemodynamic indicators** | | | |
| Heartrate, bpm | 98.00 [88.00, 114.00] | 104.00 [92.00, 116.00] | <0.001 |
| Systolic blood pressure, mmHg | 92.00 [83.00, 105.00] | 93.00 [81.00, 108.00] | 0.677 |
| Diastolic blood pressure, mmHg | 48.00 [42.00, 56.00] | 48.00 [42.00, 56.00] | 0.795 |
| Meanarterialpressure, mmHg | 62.00 [55.00, 70.00] | 62.00 [54.00, 72.00] | 0.628 |
| Lactates (mmol/L) | 2.30 [1.60, 3.10] | 2.20 [1.40, 3.40] | 0.283 |
| **Prognosis** |  |  |  |
| Vasopressor, (n (%)) | 66 (11.9) | 84 ( 15.1) | 0.136 |
| SOFA | 5.00 [3.00, 7.00] | 7.00 [6.00, 8.00] | <0.001 |
| Length of hospital stays, days (median [IQR]) | 6.61 [4.67, 11.00] | 8.73 [5.04, 15.00] | <0.001 |
| Length of icu stays, days (median [IQR]) | 2.17 [1.33, 4.23] | 3.00 [1.51, 8.00] | <0.001 |
| Hospital mortality | 59 (10.6) | 87 ( 15.7) | 0.016 |

| **Additional file table2** The 28-day prognosis analysis of patients with new ARDS was analyzed from the General Hospital of Tianjin Medical University | | | |
| --- | --- | --- | --- |
|  | 28-day-Survival group  (*n*=258) | Non-28-day-Survival group(*n*=87) | *P*-value |
| Age | 66.00 [46.25, 74.00] | 69.00 [59.00, 77.00] | 0.006 |
| Gender(male),(n (%)) | 111 (43.0) | 34 (39.1) | 0.604 |
| **Coexisting illness, (n (%))** |  |  |  |
| Chronic.pulmonary.disease | 16 ( 6.2) | 6 ( 6.9) | 1.000 |
| Immunosuppression | 78 (30.2) | 13 (14.9) | 0.008 |
| Liver.disease | 18 ( 7.0) | 6 ( 6.9) | 1.000 |
| Diabetes.disease | 92 (35.7) | 31 (35.6) | 1.000 |
| Renal.disease | 54 (20.9) | 16 (18.4) | 0.722 |
| Hypertension.disease | 137 (53.1) | 45 (51.7) | 0.922 |
| **Laboratory parameters(mean (SD))/(median [IQR])** | | | |
| White blood cell count x10ˆ^9^/L | 16.20 [11.56, 23.59] | 15.04 [11.41, 21.70] | 0.246 |
| Hemoglobin(g/dL) | 73.00 [63.25, 89.75] | 75.00 [62.00, 85.50] | 0.758 |
| Platelet (×10ˆ9 /L) | 102.00 [44.00, 172.00] | 108.00 [35.50, 164.00] | 0.808 |
| International Normalized Ratio(median [IQR]) | 1.40 [1.27, 1.64] | 1.40 [1.30, 1.69] | 0.543 |
| ProthrombinTime(median [IQR]) | 14.40 [13.40, 18.00] | 14.30 [13.40, 18.35] | 0.590 |
| Partial Thromboplastin Time (median [IQR]) | 33.95 [27.70, 42.00] | 32.70 [24.90, 40.85] | 0.200 |
| Creatinine(mg/dL) | 1.61 [0.82, 3.70] | 1.82 [1.04, 3.80] | 0.618 |
| Blood urea nitrogen (mg/dL) | 13.70 [7.10, 24.80] | 14.00 [7.65, 23.90] | 0.793 |
| **Hemodynamic indicators** |  |  |  |
| Heartrate,bpm | 110.00 [96.00, 123.00] | 110.00 [97.50, 123.00] | 0.524 |
| Systolic blood pressure,mmHg | 105.50 [89.00, 120.75] | 106.00 [89.00, 122.00] | 0.662 |
| Diastolic blood pressure,mmHg | 54.00 [45.00, 64.00] | 55.00 [40.50, 65.00] | 0.898 |
| Meanarterialpressure,mmHg | 71.00 [62.00, 82.75] | 74.00 [61.50, 82.50] | 0.902 |
| Lactates (mmol/L) | 1.70 [1.30, 2.70] | 1.60 [1.20, 2.80] | 0.762 |
| **Breathing-related indicators** |  |  |  |
| Respiration rate,bpm | 26.00 [22.00, 31.00] | 28.00 [21.50, 35.00] | 0.069 |
| FiO_2,_ (%) | 54.00 [50.00, 60.00] | 60.00 [51.50, 100.00] | <0.001 |
| SpO_2_,(%) | 95.00 [91.00, 96.00] | 91.00 [87.00, 95.50] | <0.001 |
| PaO_2_,mmHg | 102.54 [81.33, 116.58] | 89.23 [69.58, 109.03] | 0.004 |
| PaO_2_/FiO_2_ | 198.00 [140.00, 237.75] | 136.00 [84.00, 186.00] | <0.001 |
| **ARDS severity (Berlin Standard), (n (%))** |  |  | <0.001 |
| Mild | 125 (48.4) | 18 (20.7) |  |
| Moderate | 98 (38.0) | 41 (47.1) |  |
| Severe | 35 (13.6) | 28 (32.2) |  |
| SpO_2_/FiO_2_ | 173.16 [145.13, 194.00] | 144.26 [93.00, 172.73] | <0.001 |
| ROX | 6.70 [4.82, 8.50] | 5.00 [3.45, 7.25] | <0.001 |
| **Prognosis** |  |  |  |
| Vasopressor,(n (%)) | 107 (41.5) | 44 (50.6) | 0.175 |
| SOFA | 6.00 [4.00, 9.00] | 12.00 [8.00, 16.00] | <0.001 |
| APACHE II | 16.00 [13.00, 20.00] | 23.00 [18.00, 28.00] | <0.001 |
| Length of hospital stays, days (median [IQR]) | 18.00 [12.00, 25.00] | 11.00 [5.50, 20.00] | <0.001 |
| Length of icu stays, days (median [IQR]) | 10.00 [6.00, 18.00] | 10.00 [5.00, 20.00] | 0.607 |

ROX: SpO_2_ /FiO_2_/Respiratory rate; SOFA: Sequential Organ Failure Assessment; APACHE II: Acute physiology and chronic health evaluation; PaO_2_: Arterial oxygen tension; FiO_2_: fraction of inspiration O_2_; SpO_2_: pulse oximeter oxygen saturation;

| **Additional file table3** The 90-day prognosis analysis of patients with new ARDS was analyzed from the General Hospital of Tianjin Medical University | | | |
| --- | --- | --- | --- |
|  | 90-day-Survival group  (*n*=250) | Non-90-day-Survival group(*n*=95) | *P*-value |
| Age | 65.00 [46.00, 74.00] | 69.00 [59.00, 77.00] | 0.002 |
| Gender(male),(n (%)) | 108 (43.2) | 37 (38.9) | 0.553 |
| **Coexisting illness, (n (%))** |  |  |  |
| Chronic.pulmonary.disease | 16 ( 6.4) | 6 ( 6.3) | 1.000 |
| Immunosuppression | 78 (31.2) | 13 (13.7) | 0.002 |
| Liver.disease | 18 ( 7.2) | 6 ( 6.3) | 0.959 |
| Diabetes | 91 (36.4) | 32 (33.7) | 0.730 |
| Renal.disease | 51 (20.4) | 19 (20.0) | 1.000 |
| Hypertension.disease | 134 (53.6) | 48 (50.5) | 0.696 |
| **Laboratory parameters(mean (SD))/(median [IQR])** | | | |
| White blood cell count x10ˆ^9^/L | 16.20 [11.56, 23.60] | 15.04 [11.47, 21.70] | 0.250 |
| Hemoglobin(g/dL) | 73.00 [63.00, 89.00] | 76.00 [63.50, 90.50] | 0.956 |
| Platelet (×10ˆ9 /L) | 100.00 [44.00, 172.00] | 110.00 [41.50, 167.00] | 0.922 |
| International Normalized Ratio(median [IQR]) | 1.40 [1.27, 1.64] | 1.40 [1.30, 1.70] | 0.418 |
| ProthrombinTime(median [IQR]) | 14.40 [13.40, 18.00] | 14.40 [13.40, 18.35] | 0.466 |
| Partial Thromboplastin Time (median [IQR]) | 33.90 [27.38, 42.00] | 33.30 [24.90, 41.40] | 0.383 |
| Creatinine(mg/dL) | 1.62 [0.83, 3.70] | 1.73 [0.86, 3.80] | 0.808 |
| Blood urea nitrogen (mg/dL) | 14.05 [7.25, 25.23] | 14.00 [6.70, 22.90] | 0.515 |
| **Hemodynamic indicators** |  |  |  |
| Heartrate,bpm | 110.00 [96.00, 123.75] | 110.00 [97.50, 122.50] | 0.695 |
| Systolic blood pressure,mmHg | 106.00 [89.00, 120.75] | 106.00 [89.50, 122.00] | 0.519 |
| Diastolic blood pressure,mmHg | 54.00 [45.00, 64.75] | 56.00 [41.00, 65.00] | 0.978 |
| Meanarterialpressure,mmHg | 71.00 [61.25, 82.75] | 74.00 [62.00, 82.50] | 0.765 |
| Lactates (mmol/L) | 1.70 [1.30, 2.77] | 1.60 [1.20, 2.65] | 0.424 |
| **Breathing-related indicators** |  |  |  |
| Respiration rate,bpm | 26.00 [22.00, 31.00] | 29.00 [21.50, 35.00] | 0.058 |
| FiO_2,_ (%) | 53.00 [50.00, 60.00] | 60.00 [50.00, 100.00] | <0.001 |
| SpO_2_,(%) | 95.00 [91.00, 96.00] | 91.00 [88.00, 95.00] | <0.001 |
| PaO_2_,mmHg | 102.78 [82.09, 117.25] | 89.03 [69.22, 106.25] | 0.001 |
| PaO_2_/FiO_2_ | 198.00 [140.41, 238.75] | 136.00 [85.00, 186.00] | <0.001 |
| **ARDS severity (Berlin Standard), (n (%))** |  |  | <0.001 |
| Mild | 123 (49.2) | 20 (21.1) |  |
| Moderate | 95 (38.0) | 44 (46.3) |  |
| Severe | 32 (12.8) | 31 (32.6) |  |
| SpO_2_/FiO_2_ | 174.55 [145.13, 194.00] | 146.67 [93.50, 172.73] | <0.001 |
| ROX | 6.70 [4.90, 8.50] | 4.90 [3.45, 7.35] | <0.001 |
| **Prognosis** |  |  |  |
| Vasopressor,(n (%)) | 102 (40.8) | 49 (51.6) | 0.093 |
| SOFA | 6.00 [4.00, 8.75] | 12.00 [8.00, 16.00] | <0.001 |
| APACHE II | 16.00 [13.00, 20.00] | 23.00 [16.50, 27.50] | <0.001 |
| Length of hospital stays, days (median [IQR]) | 18.00 [12.00, 24.00] | 12.00 [6.00, 24.50] | <0.001 |
| Length of icu stays, days (median [IQR]) | 10.00 [6.00, 17.00] | 11.00 [5.00, 22.50] | 0.571 |

ROX: SpO_2_ /FiO_2_/Respiratory rate; SOFA: Sequential Organ Failure Assessment; APACHE II: Acute physiology and chronic health evaluation; PaO_2_: Arterial oxygen tension; FiO_2_: fraction of inspiration O_2_; SpO_2_: pulse oximeter oxygen saturation;

| **Additional file table4** The 28-day prognosis analysis of patients with new ARDS was analyzed from the eICU database | | | |
| --- | --- | --- | --- |
|  | 28-day-Survival group  (*n*=2168) | Non-28day-Survival group(n=516) | *P*-value |
| Age | 65.00 [55.00, 75.00] | 70.00 [58.00, 79.00] | <0.001 |
| Gender(male),(n (%)) | 1250 (57.7) | 296 (57.4) | 0.943 |
| **Coexisting illness, (n (%))** |  |  |  |
| Chronic.pulmonary.disease | 571 (26.3) | 133 (25.8) | 0.837 |
| Immunosuppression | 152 ( 7.0) | 52 (10.1) | 0.023 |
| Liver.disease | 109 ( 5.0) | 30 ( 5.8) | 0.539 |
| Diabetes | 409 (18.9) | 100 (19.4) | 0.837 |
| Renal.disease | 530 (24.4) | 133 (25.8) | 0.567 |
| Hypertension.disease | 500 (23.1) | 134 (26.0) | 0.180 |
| **Laboratory parameters(mean (SD))/(median [IQR])** | | | |
| White blood cell count x10ˆ^9^/L | 14.10 [10.40, 18.60] | 15.80 [10.90, 21.75] | <0.001 |
| Hemoglobin(g/dL) | 10.20 [8.60, 12.00] | 10.00 [8.40, 11.83] | 0.070 |
| Platelet (×10ˆ9 /L) | 173.00 [125.00, 228.00] | 168.00 [110.00, 234.25] | 0.115 |
| International Normalized Ratio(median [IQR]) | 1.40 [1.10, 1.60] | 1.60 [1.20, 1.60] | <0.001 |
| ProthrombinTime(median [IQR]) | 16.20 [12.40, 17.50] | 17.50 [13.30, 17.60] | <0.001 |
| Partial Thromboplastin Time (median [IQR]) | 40.60 [30.00, 40.60] | 40.60 [31.85, 40.60] | 0.001 |
| Creatinine(mg/dL) | 1.10 [0.80, 1.62] | 1.65 [1.00, 2.44] | <0.001 |
| Blood urea nitrogen (mg/dL) | 22.00 [16.00, 34.00] | 30.50 [22.00, 48.00] | <0.001 |
| **Hemodynamic indicators** | | | |
| Heartrate,bpm | 97.00 [89.00, 106.25] | 112.00 [99.00, 128.00] | <0.001 |
| Systolic blood pressure,mmHg | 166.00 [147.00, 187.00] | 169.00 [149.00, 189.25] | 0.033 |
| Diastolic blood pressure,mmHg | 84.00 [74.00, 95.25] | 89.00 [76.00, 102.00] | <0.001 |
| Meanarterialpressure,mmHg | 112.00 [100.00, 124.00] | 115.00 [102.00, 130.00] | <0.001 |
| Lactates (mmol/L) | 2.40 [1.80, 3.00] | 3.00 [2.20, 5.20] | <0.001 |
| **Breathing-related indicators** | | | |
| Respiration rate,bpm | 21.00 [14.00, 22.00] | 21.00 [14.00, 22.00] | 0.005 |
| FiO_2,_ (%) | 50.00 [40.00, 70.00] | 60.00 [50.00, 100.00] | <0.001 |
| SpO_2_,(%) | 94.00 [92.00, 98.00] | 93.00 [92.00, 97.00] | 0.086 |
| PaO_2_, mmHg | 89.00 [71.00, 116.17] | 86.00 [69.00, 116.00] | 0.081 |
| PaO_2_/FiO_2_ | 181.00 [136.00, 236.00] | 153.00 [103.75, 208.25] | <0.001 |
| **ARDS severity (Berlin Standard), (n (%))** | | | <0.001 |
| Mild | 865 (39.9) | 146 (28.3) |  |
| Moderate | 1075 (49.6) | 247 (47.9) |  |
| Severe | 228 (10.5) | 123 (23.8) |  |
| SpO_2_/FiO_2_ | 182.00 [140.00, 230.00] | 153.33 [100.00, 190.00] | <0.001 |
| ROX | 9.65 [7.10, 12.30] | 8.00 [6.30, 11.00] | <0.001 |
| **Prognosis** |  |  |  |
| Vasopressor,(n (%)) | 481 (22.2) | 235 (45.5) | <0.001 |
| SOFA | 7.00 [5.00, 9.00] | 11.00 [8.00, 14.00] | <0.001 |
| SAPS II | 54.00 [33.00, 72.00] | 48.00 [30.00, 73.00] | 0.009 |
| Length of hospital stays, days (median [IQR]) | 8.77 [5.32, 15.13] | 5.90 [2.58, 11.48] | <0.001 |
| Length of icu stays, days (median [IQR]) | 3.50 [2.00, 6.55] | 3.46 [1.83, 6.76] | 0.546 |

| **Additional file table5** The 90-day prognosis analysis of patients with new ARDS was analyzed from the eICU database | | | |
| --- | --- | --- | --- |
|  | 28-day-Survival group  (*n*=2149) | Non-28day-Survival group(*n*=535) | *P*-value |
| Age | 65.00 [55.00, 75.00] | 70.00 [58.00, 79.50] | <0.001 |
| Gender(male),(n (%)) | 1240 (57.7) | 306 (57.2) | 0.871 |
| **Coexisting illness, (n (%))** |  |  |  |
| Chronic.pulmonary.disease | 565 (26.3) | 139 (26.0) | 0.928 |
| Immunosuppression | 148 ( 6.9) | 56 (10.5) | 0.007 |
| Liver.disease | 108 ( 5.0) | 31 ( 5.8) | 0.543 |
| Diabetes.disease | 405 (18.8) | 104 (19.4) | 0.801 |
| Renal.disease | 527 (24.5) | 136 (25.4) | 0.708 |
| Hypertension.disease | 496 (23.1) | 138 (25.8) | 0.206 |
| **Laboratory parameters(mean (SD))/(median [IQR])** | | | |
| White blood cell count x10ˆ^9^/L | 14.10 [10.40, 18.50] | 15.80 [10.90, 21.98] | <0.001 |
| Hemoglobin(g/dL) | 10.20 [8.60, 12.00] | 10.00 [8.40, 11.80] | 0.043 |
| Platelet (×10ˆ9 /L) | 173.00 [125.00, 228.00] | 168.00 [110.00, 233.00] | 0.143 |
| International Normalized Ratio(median [IQR]) | 1.40 [1.10, 1.60] | 1.56 [1.20, 1.60] | <0.001 |
| ProthrombinTime(median [IQR]) | 16.10 [12.40, 17.50] | 17.50 [13.30, 17.50] | <0.001 |
| Partial Thromboplastin Time (median [IQR]) | 40.60 [30.00, 40.60] | 40.60 [32.00, 40.60] | 0.001 |
| Creatinine(mg/dL) | 1.10 [0.80, 1.62] | 1.64 [1.00, 2.42] | <0.001 |
| Blood urea nitrogen (mg/dL) | 22.00 [16.00, 34.00] | 31.00 [22.00, 48.00] | <0.001 |
| **Hemodynamic indicators** |  |  |  |
| Heartrate,bpm | 97.00 [89.00, 106.00] | 112.00 [99.00, 127.50] | <0.001 |
| Systolic blood pressure,mmHg | 166.00 [147.00, 187.00] | 169.00 [149.00, 189.00] | 0.072 |
| Diastolic blood pressure,mmHg | 84.00 [74.00, 95.00] | 88.00 [76.00, 102.00] | <0.001 |
| Meanarterialpressure,mmHg | 112.00 [100.00, 124.00] | 115.00 [102.00, 129.50] | <0.001 |
| Lactates (mmol/L) | 2.40 [1.80, 3.00] | 3.00 [2.20, 5.00] | <0.001 |
| **Breathing-related indicators** |  |  |  |
| Respiration rate,bpm | 21.00 [14.00, 22.00] | 21.00 [14.00, 22.00] | 0.001 |
| FiO_2,_ (%) | 50.00 [40.00, 70.00] | 60.00 [50.00, 100.00] | <0.001 |
| SpO_2_,(%) | 94.00 [92.00, 98.00] | 93.00 [92.00, 96.50] | 0.036 |
| PaO_2_,mmHg | 89.00 [71.00, 117.00] | 86.00 [69.00, 115.00] | 0.040 |
| PaO_2_/FiO_2_ | 182.00 [137.00, 237.00] | 153.00 [104.00, 207.50] | <0.001 |
| **ARDS severity (Berlin Standard), (n (%))** | | | <0.001 |
| Mild | 862 (40.1) | 149 (27.9) |  |
| Moderate | 1063 (49.5) | 259 (48.4) |  |
| Severe | 224 (10.4) | 127 (23.7) |  |
| SpO_2_/FiO_2_ | 182.00 [140.00, 230.00] | 153.33 [100.00, 190.00] | <0.001 |
| ROX | 9.70 [7.10, 12.30] | 8.00 [6.30, 11.00] | <0.001 |
| **Prognosis** |  |  |  |
| Vasopressor,(n (%)) | 471 (21.9) | 245 (45.8) | <0.001 |
| SOFA | 7.00 [5.00, 9.00] | 11.00 [8.00, 14.00] | <0.001 |
| SAPS II | 54.00 [33.00, 71.00] | 49.00 [30.00, 73.00] | 0.017 |
| Length of hospital stays, days (median [IQR]) | 8.71 [5.29, 14.88] | 6.08 [2.75, 12.71] | <0.001 |
| Length of icu stays, days (median [IQR]) | 3.46 [2.00, 6.50] | 3.63 [1.83, 6.86] | 0.925 |

ROX: SpO_2_ /FiO_2_/Respiratory rate; SOFA: Sequential Organ Failure Assessment; PaO_2_: Arterial oxygen tension; FiO_2_: fraction of inspiration O2; SpO_2_: pulse oximeter oxygen saturation;SAPS II: Simplified Acute Physiology Score II.

| **Additional file table 6** The 28-day prognosis analysis of patients with new ARDS was analyzed from the MIMIC IV database | | | |
| --- | --- | --- | --- |
|  | 28-day-Survival group  (*n*=1648) | Non-28-day-Survival group(*n*=161) | *P*-value |
| Age | 64.00 [54.00, 72.00] | 69.00 [57.00, 81.00] | <0.001 |
| Gender(male),(n (%)) | 519 ( 31.5) | 73 ( 45.3) | <0.001 |
| **Coexisting illness, (n (%))** |  |  |  |
| Chronic.pulmonary.disease | 229 ( 13.9) | 24 ( 14.9) | 0.815 |
| Immunosuppression | 104 ( 6.3) | 27 ( 16.8) | <0.001 |
| Liver.disease | 71 ( 4.3) | 31 ( 19.3) | <0.001 |
| Diabetes.disease | 421 ( 25.5) | 33 ( 20.5) | 0.188 |
| Renal.disease | 129 ( 7.8) | 19 ( 11.8) | 0.108 |
| Hypertension.disease | 674 ( 40.9) | 64 ( 39.8) | 0.843 |
| **Laboratory parameters(mean (SD))/(median [IQR])** | | | |
| White blood cell count x10ˆ^9^/L | 14.40 [11.40, 18.50] | 14.70 [11.80, 20.40] | 0.174 |
| Hemoglobin(g/dL) | 9.80 [8.60, 11.10] | 10.70 [9.00, 12.50] | <0.001 |
| Platelet (×10ˆ9 /L) | 146.00 [113.00, 192.00] | 171.00 [103.00, 233.00] | 0.034 |
| International Normalized Ratio(median [IQR]) | 1.30 [1.20, 1.50] | 1.40 [1.20, 2.00] | 0.001 |
| ProthrombinTime(median [IQR]) | 14.70 [13.30, 16.30] | 15.10 [12.80, 21.50] | 0.002 |
| Partial Thromboplastin Time (median [IQR]) | 31.70 [28.20, 38.50] | 35.00 [28.00, 53.30] | 0.002 |
| Creatinine(mg/dL) | 0.90 [0.70, 1.10] | 1.40 [0.90, 2.20] | <0.001 |
| Blood urea nitrogen (mg/dL) | 16.00 [12.00, 20.00] | 27.00 [19.00, 38.00] | <0.001 |
| **Hemodynamic indicators** |  |  |  |
| Heartrate,bpm | 98.00 [89.00, 110.00] | 109.00 [98.00, 126.00] | <0.001 |
| Systolic blood pressure,mmHg | 89.00 [82.00, 97.00] | 88.00 [76.00, 102.00] | 0.246 |
| Diastolic blood pressure,mmHg | 46.00 [41.00, 51.00] | 44.00 [38.00, 52.00] | 0.046 |
| Meanarterialpressure,mmHg | 59.00 [54.00, 64.00] | 57.00 [48.00, 65.00] | 0.003 |
| Lactates (mmol/L) | 2.40 [1.80, 3.20] | 3.20 [1.90, 6.30] | <0.001 |
| **Breathing-related indicators** | | | |
| Respiration rate,bpm | 26.00 [23.00, 29.00] | 29.00 [24.00, 32.00] | <0.001 |
| FiO_2,_ (%) | 100.00 [50.00, 100.00] | 65.00 [50.00, 100.00] | <0.001 |
| SpO_2_,(%) | 94.00 [92.00, 95.00] | 93.00 [87.00, 96.00] | 0.047 |
| PaO_2_,mmHg | 119.00 [101.00, 171.00] | 96.00 [76.00, 121.00] | <0.001 |
| PaO_2_/FiO_2_ | 168.00 [117.00, 222.50] | 136.00 [96.00, 180.00] | <0.001 |
| **ARDS severity (Berlin Standard), (n (%))** | | | <0.001 |
| Mild | 528 ( 32.0) | 30 ( 18.6) |  |
| Moderate | 990 ( 60.1) | 87 ( 54.0) |  |
| Severe | 130 ( 7.9) | 44 ( 27.3) |  |
| SpO_2_/FiO_2_ | 168.00 [117.00, 222.50] | 136.67 [97.00, 186.00] | <0.001 |
| ROX | 6.45 [4.62, 8.54] | 4.92 [3.18, 7.25] | <0.001 |
| **Prognosis** |  |  |  |
| Vasopressor,(n (%)) | 1648 (100.0) | 161 (100.0) | 1.000 |
| SOFA | 4.00 [3.00, 6.00] | 8.00 [5.00, 12.00] | <0.001 |
| SAPS II | 31.00 [25.00, 39.00] | 47.00 [37.00, 60.00] | <0.001 |
| Length of hospital stays, days (median [IQR]) | 8.71 [5.29, 14.88] | 6.08 [2.75, 12.71] | <0.001 |
| Length of icu stays, days (median [IQR]) | 3.46 [2.00, 6.50] | 3.63 [1.83, 6.86] | 0.925 |

ROX: SpO_2_ /FiO_2_/Respiratory rate; SOFA: Sequential Organ Failure Assessment; PaO_2_: Arterial oxygen tension; FiO_2_: fraction of inspiration O2; SpO_2_: pulse oximeter oxygen saturation; SAPS II: Simplified Acute Physiology Score II.

| **Additional file table7** The 90-day prognosis analysis of patients with new ARDS was analyzed from the MIMIC IV database | | | |
| --- | --- | --- | --- |
|  | 90-day-Survival group  (*n*=1646) | Non-90-day-Survival group(*n*=163) | *P*-value |
| Age | 64.00 [54.00, 72.00] | 69.00 [57.00, 81.00] | <0.001 |
| Gender(male),(n (%)) | 518 ( 31.5) | 74 ( 45.4) | <0.001 |
| **Coexisting illness, (n (%))** |  |  |  |
| Chronic.pulmonary.disease | 229 ( 13.9) | 24 ( 14.7) | 0.868 |
| Immunosuppression | 104 ( 6.3) | 27 ( 16.6) | <0.001 |
| Liver.disease | 70 ( 4.3) | 32 ( 19.6) | <0.001 |
| Diabetes.disease | 421 ( 25.6) | 33 ( 20.2) | 0.161 |
| Renal.disease | 128 ( 7.8) | 20 ( 12.3) | 0.065 |
| Hypertension.disease | 673 ( 40.9) | 65 ( 39.9) | 0.868 |
| **Laboratory parameters(mean (SD))/(median [IQR])** | | | |
| White blood cell count x10ˆ^9^/L | 14.40 [11.40, 18.50] | 14.60 [11.70, 20.25] | 0.213 |
| Hemoglobin(g/dL) | 9.80 [8.60, 11.10] | 10.70 [8.95, 12.50] | <0.001 |
| Platelet (×10ˆ9 /L) | 146.00 [113.25, 192.00] | 171.00 [102.00, 231.00] | 0.043 |
| International Normalized Ratio(median [IQR]) | 1.30 [1.20, 1.50] | 1.40 [1.20, 2.00] | 0.001 |
| ProthrombinTime(median [IQR]) | 14.70 [13.30, 16.30] | 15.10 [12.85, 21.65] | 0.002 |
| Partial Thromboplastin Time (median [IQR]) | 31.70 [28.22, 38.50] | 35.00 [27.95, 55.70] | 0.002 |
| Creatinine(mg/dL) | 0.90 [0.70, 1.10] | 1.40 [0.90, 2.20] | <0.001 |
| Blood urea nitrogen (mg/dL) | 16.00 [12.00, 20.00] | 27.00 [19.00, 39.50] | <0.001 |
| **Hemodynamic indicators** |  |  |  |
| Heartrate,bpm | 98.00 [89.00, 110.00] | 109.00 [97.50, 126.00] | <0.001 |
| Systolic blood pressure,mmHg | 89.00 [82.00, 97.00] | 88.00 [75.50, 102.50] | 0.244 |
| Diastolic blood pressure,mmHg | 46.00 [41.00, 51.00] | 43.00 [37.00, 52.00] | 0.025 |
| Meanarterialpressure,mmHg | 59.00 [54.00, 64.00] | 57.00 [48.00, 64.50] | 0.002 |
| Lactates (mmol/L) | 2.40 [1.80, 3.20] | 3.20 [1.85, 6.35] | <0.001 |
| **Breathing-related indicators** | | |  |
| Respiration rate,bpm | 26.00 [23.00, 29.00] | 29.00 [24.00, 32.00] | <0.001 |
| FiO_2,_ (%) | 100.00 [50.00, 100.00] | 65.00 [50.00, 100.00] | <0.001 |
| SpO_2_,(%) | 94.00 [92.00, 95.00] | 93.00 [87.00, 96.00] | 0.051 |
| PaO_2_,mmHg | 119.00 [101.00, 171.00] | 96.00 [76.50, 120.50] | <0.001 |
| PaO_2_/FiO_2_ | 168.00 [117.00, 222.50] | 135.56 [96.50, 179.00] | <0.001 |
| **ARDS severity (Berlin Standard), (n (%))** | | | <0.001 |
| Mild | 528 ( 32.1) | 30 ( 18.4) |  |
| Moderate | 989 ( 60.1) | 88 ( 54.0) |  |
| Severe | 129 ( 7.8) | 45 ( 27.6) |  |
| SpO_2_/FiO_2_ | 168.00 [117.00, 222.50] | 136.00 [97.67, 183.00] | <0.001 |
| ROX | 6.46 [4.63, 8.54] | 4.88 [3.18, 7.10] | <0.001 |
| **Prognosis** | | | |
| Vasopressor,(n (%)) | 1646 (100.0) | 163 (100.0) | <0.001 |
| SOFA | 4.00 [3.00, 6.00] | 8.00 [5.00, 12.00] | <0.001 |
| SAPS II | 31.00 [25.00, 39.00] | 47.00 [37.00, 59.50] | <0.001 |
| Length of hospital stays, days (median [IQR]) | 5.94 [4.58, 8.18] | 2.69 [1.60, 5.15] | <0.001 |
| Length of icu stays, days (median [IQR]) | 1.77 [1.25, 2.74] | 2.16 [1.58, 3.91] | <0.001 |

ROX: SpO_2_ /FiO_2_/Respiratory rate; SOFA: Sequential Organ Failure Assessment; PaO_2_: Arterial oxygen tension; FiO_2_: fraction of inspiration O2; SpO_2_: pulse oximeter oxygen saturation;SAPS II: Simplified Acute Physiology Score II.

| **Additional file table8** Univariate and multivariate analysis of 28-day survival in patients with ARDS in three database. | | | | | | |
| --- | --- | --- | --- | --- | --- | --- |
| **characteristic** | Univariate analysis | | | Multivariate analysis | | |
|  | HR | 95%CI | *P* | HR | 95%CI | *P* |
| Age | 1.02 | 1.01-1.02 | <0.01 | 1.01 | 1.01 - 1.02 | <0.010 |
| Gender | 1.13 | 0.98 - 1.3 | 0.103 |  |  |  |
| **Coexisting illness, (n (%))** |  |  |  |  |  |  |
| Chronic.pulmonary.disease | 1.02 | 0.86 - 1.21 | 0.838 |  |  |  |
| Immunosuppression | 1.24 | 0.99 - 1.54 | 0.057 |  |  |  |
| Liver.disease | 1.68 | 1.31 - 2.15 | <0.01 | 1.17 | 0.90 - 1.51 | 0.250 |
| Diabetes | 0.92 | 0.77 - 1.09 | 0.348 |  |  |  |
| Renal.disease | 1.12 | 0.95 - 1.33 | 0.186 |  |  |  |
| Hypertension.disease | 0.97 | 0.83 - 1.13 | 0.667 |  |  |  |
| **Laboratory parameters(mean (SD))/(median [IQR])** |  |  |  |  |  |  |
| White blood cell count | 1.01 | 1.01 - 1.02 | 0.001 | 1.00 | 0.99 - 1.00 | 0.320 |
| Hemoglobin | 1.00 | 1.00 -1.00 | 0.355 | 1.10 | 0.93 - 1.29 | 0.260 |
| Platelet | 1.00 | 1.00-1.00 | 0.768 |  |  |  |
| International Normalized Ratio(median | 1.26 | 1.2 - 1.33 | <0.01 | 1.18 | 1.07 - 1.30 | <0.001 |
| ProthrombinTime(median | 1.02 | 1.01 - 1.02 | <0.01 | 0.99 | 0.98 - 1.00 | 0.120 |
| Partial Thromboplastin Time (median | 1.01 | 1.00 - 1.01 | <0.01 | 1.00 | 1.00-1.00 | 0.940 |
| Creatinine | 1.08 | 1.05 - 1.11 | <0.01 | 0.96 | 0.92 - 1.00 | 0.050 |
| Blood urea nitrogen | 1.02 | 1.01 - 1.02 | <0.01 | 1.00 | 1.00 - 1.01 | 0.010 |
| **Hemodynamic indicators** |  |  |  |  |  |  |
| Heart rate> 100bpm | 2.57 | 2.2 - 3.01 | <0.01 | 1.76 | 1.49 - 2.07 | <0.01 |
| Low blood pressure | 1.21 | 1.01 - 1.45 | 0.036 | 1.12 | 0.9 - 1.39 | 0.320 |
| Lactates | 1.15 | 1.13 - 1.17 | <0.01 | 1.10 | 1.08 - 1.12 | <0.01 |
| **Breathing-related indicators** |  |  |  |  |  |  |
| Respiration rate,bpm | 1.00 | 0.99 - 1.01 | 0.396 |  |  |  |
| FiO_2_ | 1.01 | 1.00 - 1.01 | <0.01 |  |  |  |
| SpO_2_ | 0.97 | 0.95 - 0.98 | <0.01 | 0.99 | 0.98 - 1.00 | 0.090 |
| PaO_2_ | 1.00 | 0.99 - 1.00 | <0.01 | 1.00 | 0.99 - 1.00 | 0.900 |
| PaO_2_/FiO_2_ | 0.99 | 0.99 - 1.00 | <0.01 | 1.00 | 0.99 - 1.00 | 0.840 |
| **ARDS severity (Berlin Standard), (n (%))** | 1.66 | 1.49 - 1.84 | <0.01 |  |  |  |
| Mild |  |  |  |  |  |  |
| Moderate |  |  |  |  |  |  |
| Severe |  |  |  |  |  |  |
| SpO_2_/FiO_2_ | 0.99 | 0.99 - 0.99 | <0.01 | 1.00 | 0.99 - 1 | 0.010 |
| ROX | 0.93 | 0.91 - 0.95 | <0.01 | 0.99 | 0.95 - 1.03 | 0.560 |
| **Prognosis** |  |  |  |  |  |  |
| Vasopressor,(n (%)) | 1.93 | 1.66 - 2.24 | <0.01 |  |  |  |
| SOFA | 1.20 | 1.19 - 1.22 | <0.01 | 1.16 | 1.14 - 1.18 | <0.010 |

ROX: SpO_2_ /FiO_2_/Respiratory rate; SOFA: Sequential Organ Failure Assessment; PaO_2_: Arterial oxygen tension; FiO_2_: fraction of inspiration O2; SpO_2_: pulse oximeter oxygen saturation;

| **Additional file table9** Comparison of SOFA scores, APACHE II scores, and SAPS II scores in databases and new and Berlin ARDS diagnostic criteria | | | | | |
| --- | --- | --- | --- | --- | --- |
| General Hospital of Tianjin Medical University database | | | | | |
|  | Berlin-criteria ARDS | HFNO ARDS | Newly criteria ARDS | P | Figure |
| SOFA | [7.00 (5.00, 12.00)] | [7.00 (4.00, 9.00)] | [7.00 (5.00, 11.00)] | 0.239 | Figure 3 A |
| APACHE II | [18.00 (14.00, 23.00)] | [16.00 (13.00, 18.75)] | [17.00 (13.00, 22.00)] | 0.016 | Figure 3 B |
| eICU database | | | | | |
|  | Berlin-criteria ARDS | HFNO ARDS | Newly criteria ARDS | P | Figure |
| SOFA | [7.00 (5.00, 10.00)] | 6.00 [4.00, 7.75] | [7.00 (5.00, 10.00)] | 0.01 | Figure 3 C |
| SAPS II | 53.00 [31.00, 72.00] | 49.00 [30.75, 62.00] | 53.00 [31.00, 72.00] | 0.454 | Figure 3 D |
| MIMIC-IV database | | | | | |
|  | Berlin-criteria ARDS | HFNO ARDS | Newly criteria ARDS | P | Figure |
| SOFA | [4.00 (3.00, 6.00)] | [4.00 (3.00, 5.00)] | [4.00 (3.00, 6.00)] | 0.619 | Figure 3 E |
| SAPS II | [33.00 (26.00, 41.00)] | [31.00 (25.00, 38.00)] | [33.00 (26.00, 41.00)] | 0.095 | Figure 3 F |

**Additional file Fig S2** The SOFA score and the APACHE II、SAPS II score predict the ROC curve the prognosis of newly criteria ARDS patients


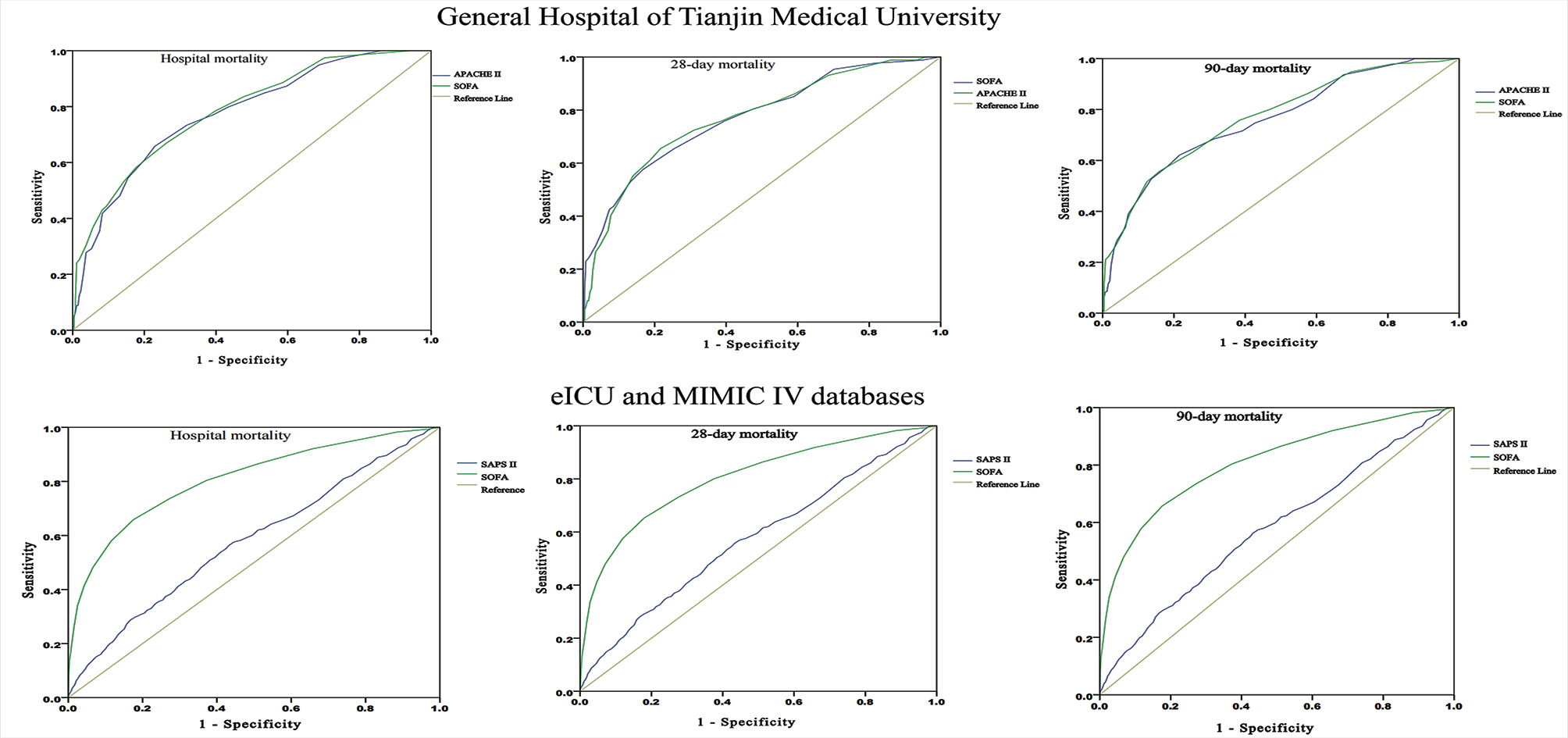


In the General Hospital of Tianjin Medical University, APACHE II for hospital mortality prediction: AUC: 0.776 95% CI: 0.718-0.883, Specificity: 77.1%, Sensitivity: 65.8%. SOFA for hospital mortality prediction: AUC: 0.785 95% CI: 0.728-0.842, Specificity:73.7%, Sensitivity: 67.1%. APACHE II for 28-day mortality:AUC: 0.771 95% CI: 0.713-0.829, Specificity: 78.3%, Sensitivity: 65.5%; SOFA for 28-day mortality:AUC:0.771, 95% CI:0.713-0.830, Specificity: 83.3%, Sensitivity: 57.5%; APACHE II for 90-day mortality:AUC: 0.758, 95% CI: 0.701-0.806, Specificity: 73.9%, Sensitivity: 62.1%; SOFA for 90-day mortality:AUC:0.769, 95% CI:0.712-0.825, Specificity:84.0%, Sensitivity: 55.8%.

In MIMIC IV and EICU database, SAPS II for hospital mortality prediction: AUC: :0.580, 95% CI: 0.556-0.603, Specificity: 56.8%, Sensitivity: 56.3%. SOFA for hospital mortality prediction: AUC: 0.808 95% CI: 0.788-0.827, Specificity:82.5%, Sensitivity: 65.9%. SAPS II for 28-day mortality:AUC: 0.575, 95% CI: 0.551-0.599, Specificity: 83.1%, Sensitivity: 28.2%; SOFA for 28-day mortality:AUC:0.802, 95% CI:0.783-0.822, Specificity: 82.0%, Sensitivity: 65.3%; SAPS II for 90-day mortality:AUC: 0.578, 95% CI: :0.555-0.602, Specificity: 56.8%, Sensitivity: 56.3%; SOFA for 90-day mortality:AUC:0.806, 95% CI::0.787-0.826, Specificity:82.0%, Sensitivity: 65.9%.

**Additional Fig S3**


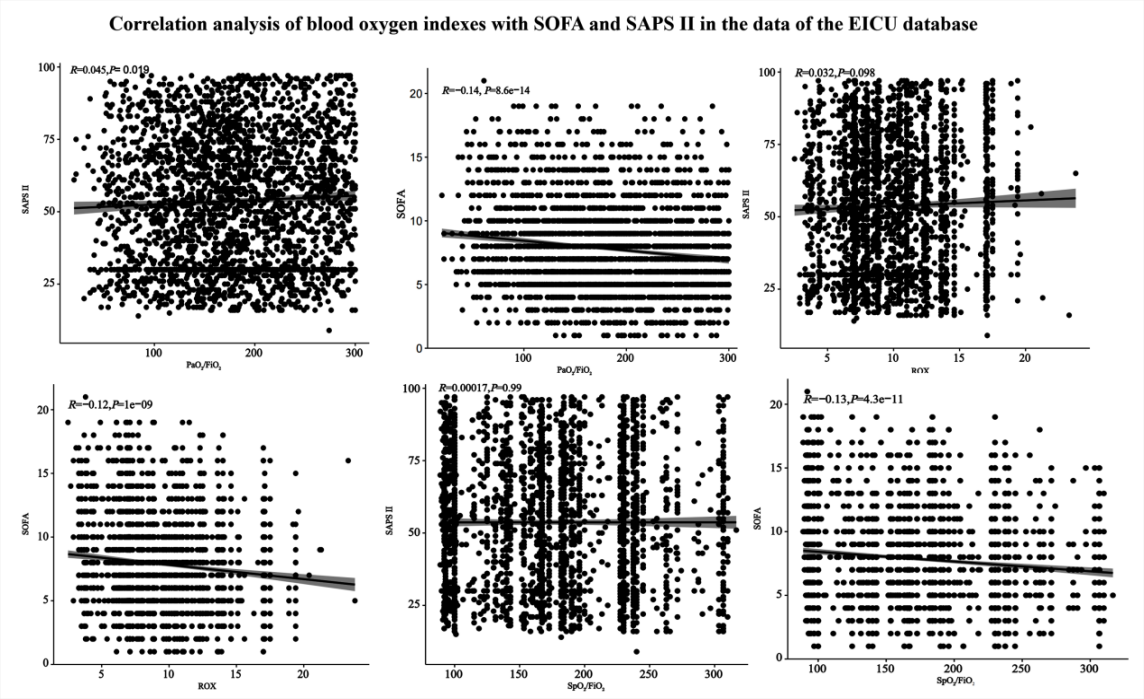


PaO_2_/FiO_2_, SpO_2_/FiO_2_, and ROX were negatively correlated with SOFA.

**Additional Fig S4**


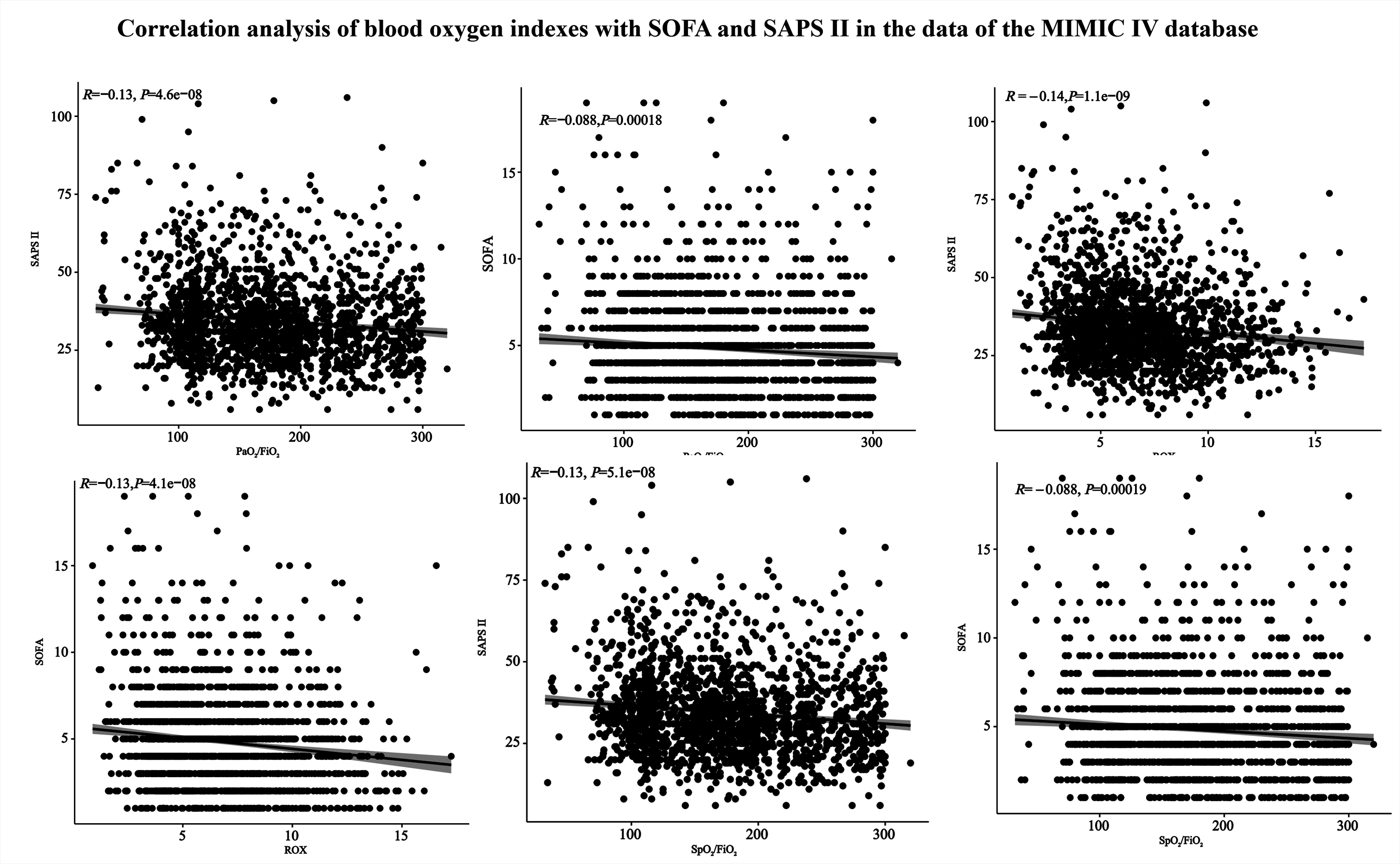


PaO_2_/FiO_2_, SpO_2_/FiO_2_, and ROX were negatively correlated with SOFA and SAPS II.

**Additional file Fig S5: Multivariate correlation analysis plot**


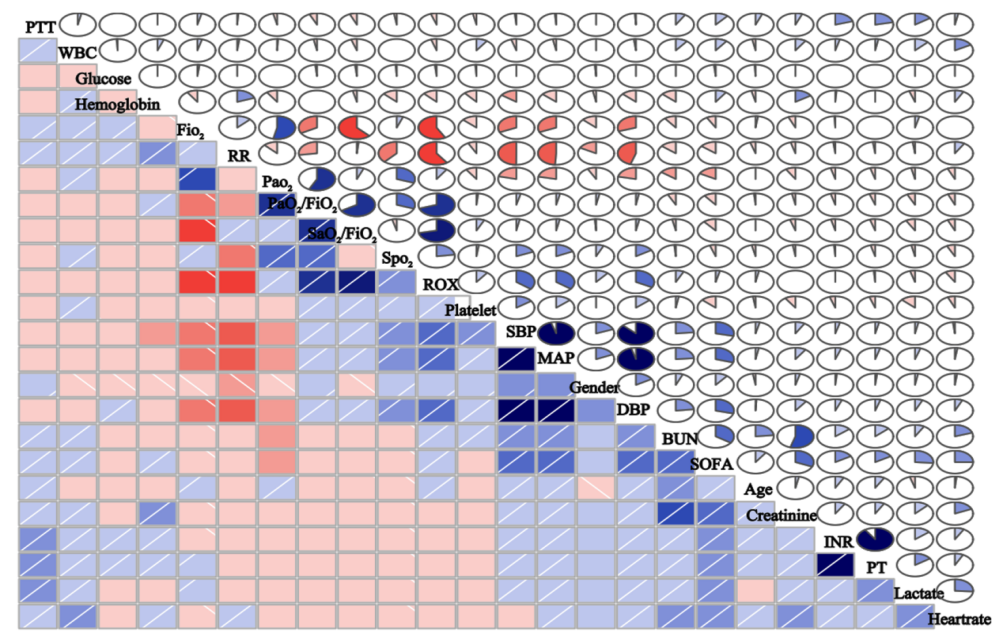


WBC:White blood cell count; INR:International Normalized Ratio;PT:ProthrombinTime;PTT:Partial Thromboplastin Time;BUN:Blood urea nitrogen;ROX: SpO_2_ /FiO_2_/RR; SOFA: Sequential Organ Failure Assessment; PaO_2_: Arterial oxygen tension; FiO_2_: fraction of inspiration O_2_; SpO_2_: pulse oximeter oxygen saturation.SBP:Systolic blood pressure;DBP:Diastolic blood pressure;RR:Respiratory rate.

**Additional file Fig S6 Missing values for variables in the data**


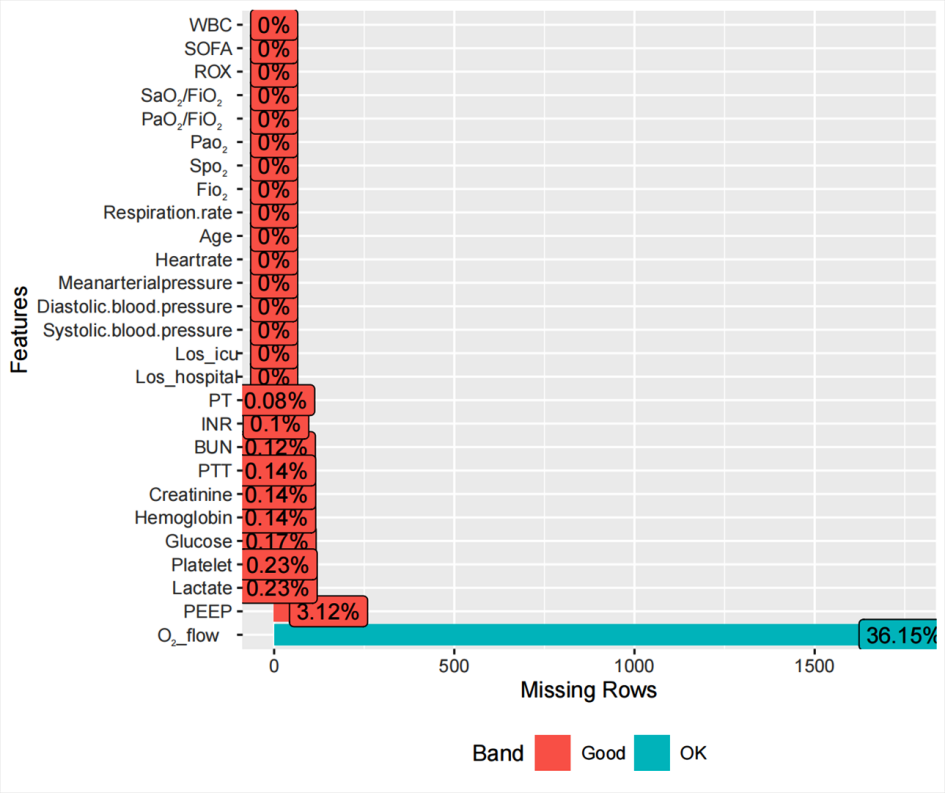


**Additional file Fig S7** The distribution of variables in the data


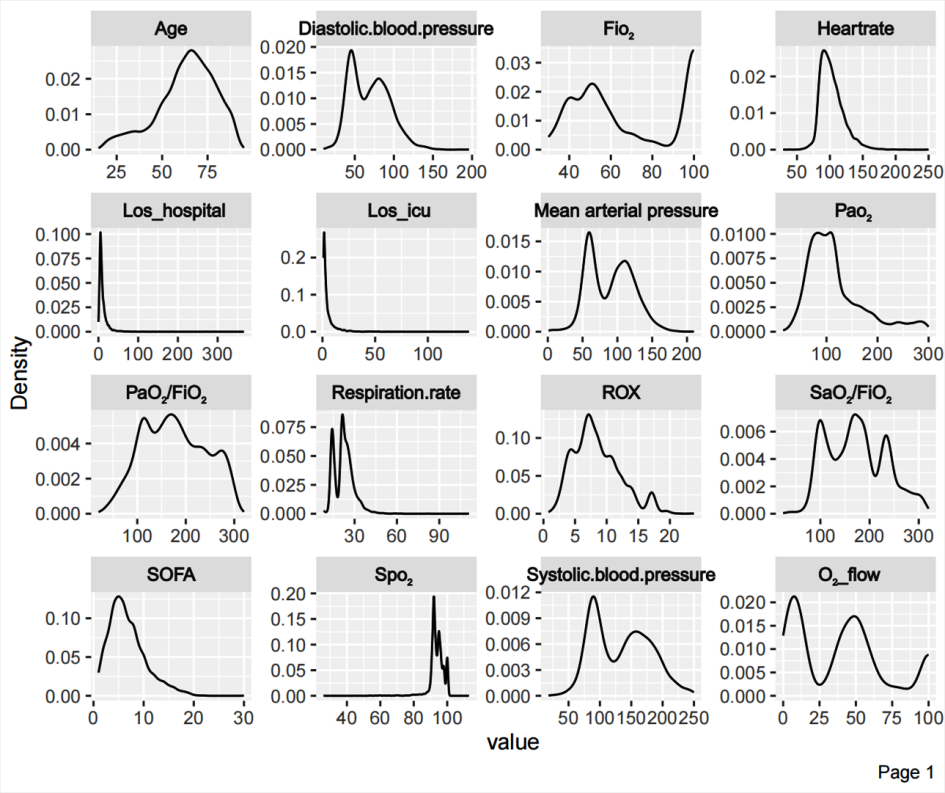


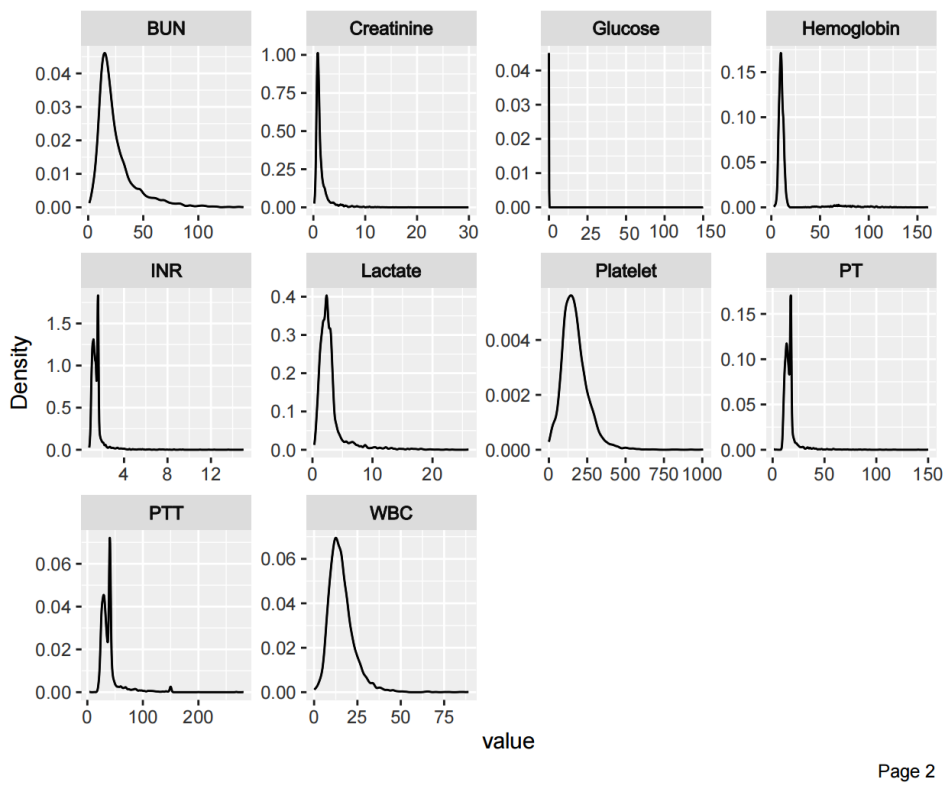


| **Additional file table10** | **Direction of abnormal values and distribution transformation** |
| --- | --- |
| Variable | Abnormal value extractor direction |
| Age | - |
| Gender | - |
| Heart rate | max |
| Respiratory rate | max |
| Systolic blood pressure | min |
| Diastolic blood pressure | min |
| Meanarterialpressure | min |
| White blood cell count | max |
| Hemoglobin | min |
| Platelet | min |
| International Normalized Ratio | max |
| ProthrombinTime | max |
| Partial Thromboplastin Time | max |
| Creatinine | max |
| Blood urea nitrogen | max |
| Lactates | max |
| FiO_2_ | min |
| SpO_2_ | min |
| PaO_2_ | min |
| FiO_2_ | max |
